# KO*t*-Bu-promoted selective ring-opening *N*-alkylation of 2-oxazolines to access 2-aminoethyl acetates and *N*-substituted thiazolidinones

**DOI:** 10.3762/bjoc.16.44

**Published:** 2020-03-25

**Authors:** Qiao Lin, Shiling Zhang, Bin Li

**Affiliations:** 1 School of Biotechnology and Health Sciences, Wuyi University, Jiangmen 529020, Guangdong Province, P.R. China

**Keywords:** *N*-alkylation, oxazolines, potassium *tert*-butoxide, ring opening, thiazolidinones

## Abstract

An efficient and simple KO*t*-Bu-promoted selective ring-opening *N*-alkylation of 2-methyl-2-oxazoline or 2-(methylthio)-4,5-dihydrothiazole with benzyl halides under basic conditions is described for the first time. The method provides a convenient and practical pathway for the synthesis of versatile 2-aminoethyl acetates and *N*-substituted thiazolidinones with good functional group tolerance and selectivity. KO*t*-Bu not only plays an important role to promote this ring-opening *N*-alkylation, but also acts as an oxygen donor.

## Introduction

**Scheme 1:**
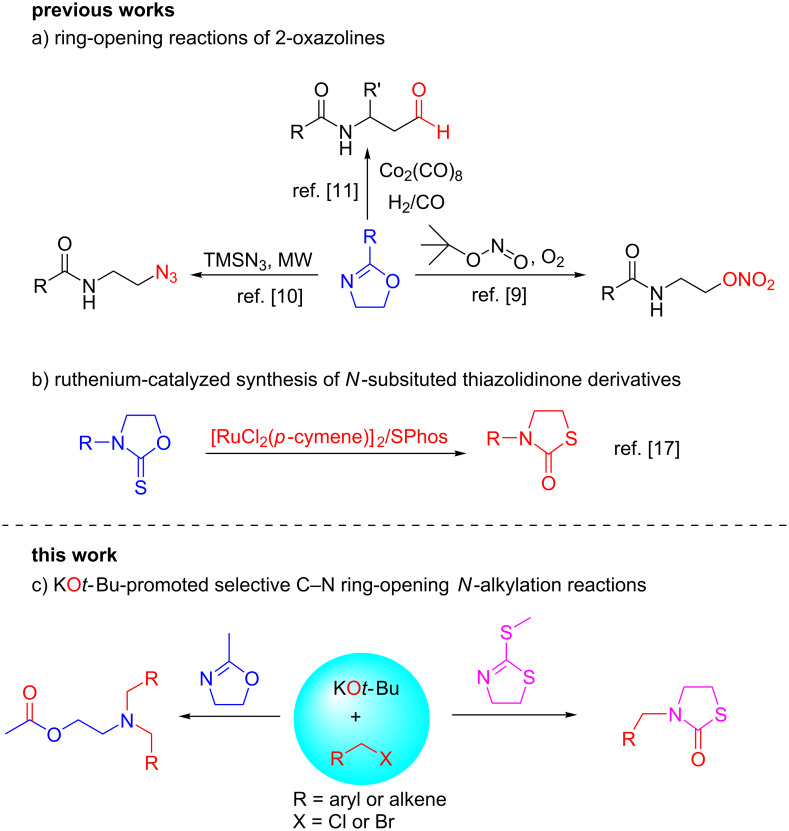
Comparison of different ring-opening reactions of 2-oxazolines and thiazolidinones synthesis.

2-Oxazolines are important structural units in pharmaceutical applications and efficient ligands in coordination chemistry, and also valuable protecting or directing groups in catalysis [1–3]. 2-Oxazolines are a readily stable class of heterocycles resistant to a range of nucleophiles, bases, or radicals [4–5], which can be easily generated from amino alcohols and carboxylic acids, and from alkenes or epoxides as substrates via alternative synthetic procedures [6]. However, under acidic conditions, oxazolines transform into β-substituted carboxamides through nucleophilic ring opening with S_N_2 attack at the C5 position of the ring [7–8]. Recently, Guo’s group developed an efficient method for the synthesis of β-nitrate ester carboxamides using *tert*-butyl nitrite as the nitro source and oxygen as the oxidant through the ring opening of 2-oxazolines [9] (Scheme 1a). Kappe reported a two-step continuous-flow synthesis of *N*-(2-aminoethyl)acylamides through ring opening/hydrogenation of oxazolines with TMSN_3_ as the azide source [10] (Scheme 1a). Coates described a Co_2_(CO)_8_-catalyzed ring-opening hydroformylation of oxazolines for the synthesis of β-amidoaldehydes [11] (Scheme 1a). However, the ring-opening *N*-alkylation of 2-oxazolines to produce 2-aminoethyl acetate derivatives under basic conditions has not been reported.

Thiazolidinone derivatives are important moieties in functional materials and natural products [12–14], such as latrunculin that has been obtained from the sponge *Cacospongia mycofijiensis* [15]. Convenient syntheses of thiazolidinone derivatives are highly attractive to synthetic chemists. However, only a few examples are reported for the synthesis of *N*-substituted thiazolidinones although the synthetic method of thiazolidinone was described in 1993 [16]. Frost and co-workers explored an efficient ruthenium-catalyzed *O-*to-*S-*alkyl migration of *N*-alkyloxazolidine-2-thiones to synthesize thiazolidinone derivatives through a Barton–McCombie pathway in 2015 [17] (Scheme 1b).

Recently, potassium *tert*-butoxide has been shown to be an efficient promoter for C–C-bond formation reactions [18–22]. However, only few reports described C–N-bond cross-coupling reactions using potassium *tert*-butoxide as promoter. Wu developed an efficient protocol for the KO*t*-Bu-promoted synthesis of 1-aminoisoquinolines from 2-methylbenzonitriles and benzonitriles [23], and the carbonylative cyclization of propargylic amines with selenium under CO gas-free conditions [24]. Based on our continuing interest in developing new transformation methodologies of oxazolines [25], herein, we report a simple KO*t*-Bu-promoted selective ring-opening *N*-alkylation of 2-methyl-2-oxazolines and 2-(methylthio)-4,5-dihydrothiazole with benzyl halides, leading to 2-aminoethyl acetates and *N*-substituted thiazolidinone derivatives under mild conditions (Scheme 1c).

## Results and Discussion

To test this ring-opening *N*-alkylation of 2-oxazoline, benzyl bromide (**1a**) and 2-methyl-2-oxazoline (**2**) were chosen as the model substrates for the reaction in the presence of 20 mol % of CuBr_2_, 2 equiv of KO*t*-Bu in CH_3_CN at 100 °C for 16 h, and a full conversion to the 2-aminoethyl acetate product **3a** was obtained (Table 1, entry 1). By changing the copper salt to CuBr or CuI, similar results were detected under the same conditions (Table 1, entries 2 and 3). Surprisingly, when this reaction was performed without copper salts and decreasing the temperature to 50 °C in CH_3_CN, still a 99% GC yield of the desired product **3a** was obtained (Table 1, entries 4 and 5). These results revealed that the copper salt is not necessary for this ring-opening *N*-alkylation reaction to take place. Next we evaluated several solvents, including toluene, EtOH, THF, H_2_O, CH_2_Cl_2_ and dimethyl carbonate (DMC). Among them, DMC afforded the product **3a** in an excellent yield and as greener solvent compared to CH_3_CN, it was selected as the best solvent for this ring-opening *N*-alkylation (Table 1, entry 11). When replacing KO*t*-Bu by other potassium salts such as KOH, KOAc and PhCOOK the product yield significantly dropped (Table 1, entries 13–15). Also, decreasing the temperature to room temperature or the amount of KO*t-*Bu to 0.5 equiv led to lower yields (Table 1, entries 16 and 17). No product was obtained when the reaction was repeated in the absence of a KO*t*-Bu as base (Table 1, entry 18), demonstrating that KO*t*-Bu plays an important role for promoting this ring-opening *N*-alkylation. Finally, performing the reaction with 1.0 equiv of KO*t*-Bu in DMC at 50 °C for 16 h, was found to be the optimized conditions.

**Table 1:** Optimization of the KO*t*-Bu-promoted selective ring-opening *N*-alkylation of 2-methyl-2-oxazoline with benzyl bromide.^a^

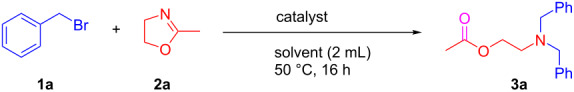					

Entry	Catalyst (mol %)	Base (equiv)	Solvent	Temperature (°C)	GC yield (%)

1	CuBr_2_ (20)	KO*t*-Bu (2)	CH_3_CN	100	99
2	CuBr (20)	KO*t*-Bu (2)	CH_3_CN	100	99
3	CuI (20)	KO*t*-Bu (2)	CH_3_CN	100	99
4	–	KO*t*-Bu (2)	CH_3_CN	100	99
5	–	KO*t*-Bu (2)	CH_3_CN	50	99
6	–	KO*t*-Bu (2)	toluene	50	58
7	–	KO*t*-Bu (2)	EtOH	50	25
8	–	KO*t*-Bu (2)	THF	50	66
9	–	KO*t*-Bu (2)	H_2_O	50	–
10	–	KO*t*-Bu (2)	CH_2_Cl_2_	50	98
11	–	KO*t*-Bu (2)	DMC	50	98
12	–	KO*t*-Bu (1)	DMC	50	97
13	–	KOH (1)	DMC	50	76
14	–	KOAc (1)	DMC	50	64
15	–	PhCO_2_K (1)	DMC	50	70
16	–	KO*t*-Bu (0.5)	DMC	50	48
17	–	KO*t*-Bu (0.5)	DMC	rt	31
18	–	–	DMC	50	–

^a^KO*t-*Bu, 2-methyl-2-oxazoline (0.5 mmol), benzyl bromide (1.0 mmol), solvent (2 mL), under air for 16 h.

**Scheme 2:**
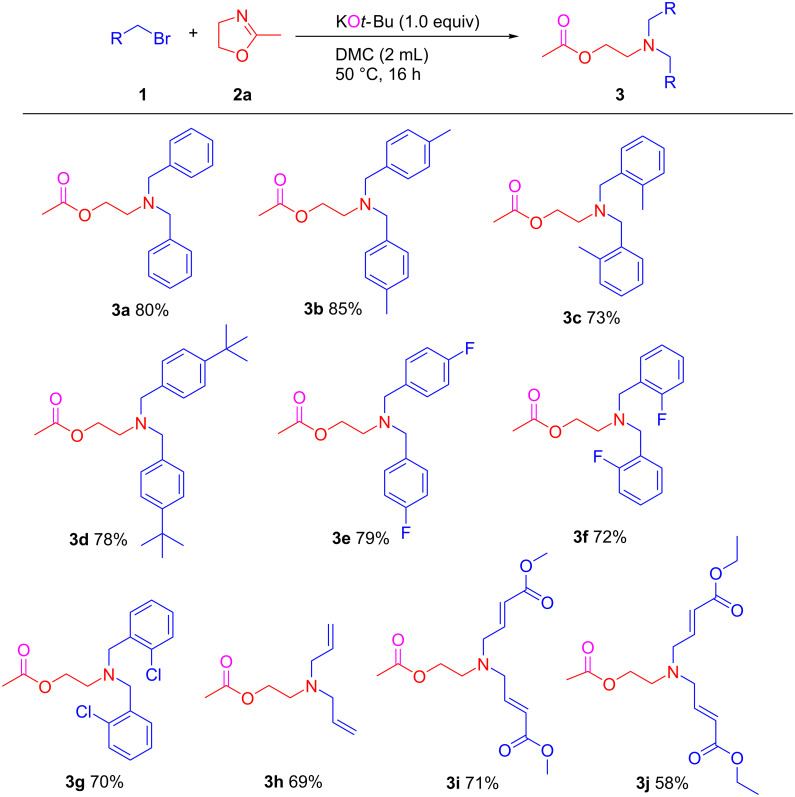
KO*t*-Bu-promoted selective ring-opening *N*-alkylation of 2-methyl-2-oxazoline with benzyl bromides. Conditions: KO*t*-Bu (0.5 mmol), 2-methyl-2-oxazoline (0.5 mmol), benzyl bromides (1.0 mmol) in DMC (2 mL) at 50 °C, under air for 16 h.

Next, the scope and limitations of this KO*t*-Bu-promoted ring-opening *N*-alkylation from alkyl bromides with 2-methyl-2-oxazolines were explored using the optimized conditions. As shown in Scheme 2, various benzyl bromides bearing -Me, -*t*-Bu, -F, and -Cl groups were applied in the synthesis and afforded the tertiary amines **3a**–**g** in 70–85% yield, respectively. Notably, the steric and inductive effects of the substituents did not hamper this ring-opening *N*-alkylation. Allyl bromide (**1h**) successfully reacted with 2-methyl-2-oxazoline and produced the corresponding product **3h** in good yield (69%). More importantly, bromide-containing enoate derivatives **1i** and **1j** were easily transferred to the corresponding ring-opened *N*-alkylated triesters **3i** and **3j** containing two C=C bonds which could be important as precursors for double Michael additions, and were isolated in 71% and 58% yields.

**Scheme 3:**
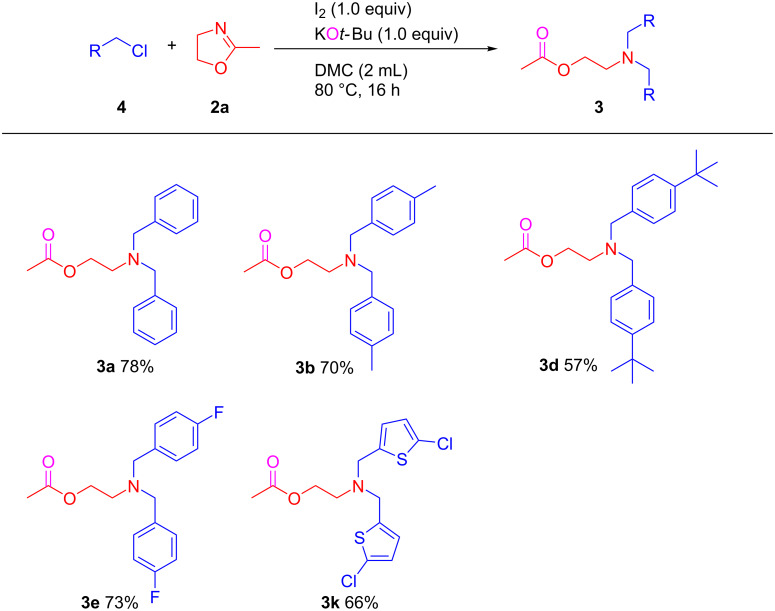
KO*t*-Bu-promoted selective ring-opening *N*-alkylation of 2-methyl-2-oxazoline with benzyl chlorides. Conditions: KO*t*-Bu (0.5 mmol), 2-methyl-2-oxazoline (0.5 mmol), benzyl chlorides (1.0 mmol), I_2_ (0.5 mmol) in DMC (2 mL), at 80 °C, under air for 16 h.

The above described synthetic system has been evaluated for the ring-opening *N*-alkylation with benzyl chloride derivatives **4** under similar conditions but at 80 °C, as the chlorides are expected to be less reactive than the corresponding benzyl bromides (Scheme 3). Only 26% yield of 2-aminoethyl acetate compound **3a** were observed, however, the addition of 1.0 equiv of I_2_ allowed to increase the conversion up to 95% and product **3a** was isolated in 78% yield. Other benzyl chlorides bearing -Me, -*t*-Bu, and -F groups in the *para*-position were applied to generate corresponding products **3b**, **3d**, and **3e** in moderate to good yields. Furthermore, the reaction proceeded well with heterocycle-containing chlorides such as 2-chloro-5-(chloromethyl)thiophene (**4e**), leading to 2-aminoethyl acetate product **3k** in 66% yield which has potential as bifunctional monomer for polymerization.

**Scheme 4:**
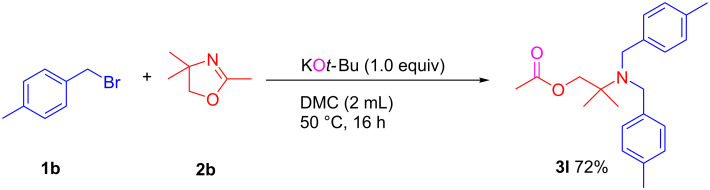
KO*t*-Bu-promoted selective ring-opening *N*-alkylation of 2,4,4-trimethyl-4,5-dihydrooxazole (**2b**) with 4-methylbenzyl bromide (**1b**).

Furthermore, oxazole derivative 2,4,4-trimethyl-4,5-dihydrooxazole (**2b**) was also examined in the KO*t*-Bu-promoted ring-opening *N*-alkylation with 4-methylbenzyl bromide, which successfully led to the corresponding product **3l** in 72% isolated yield as shown in Scheme 4.

**Scheme 5:**
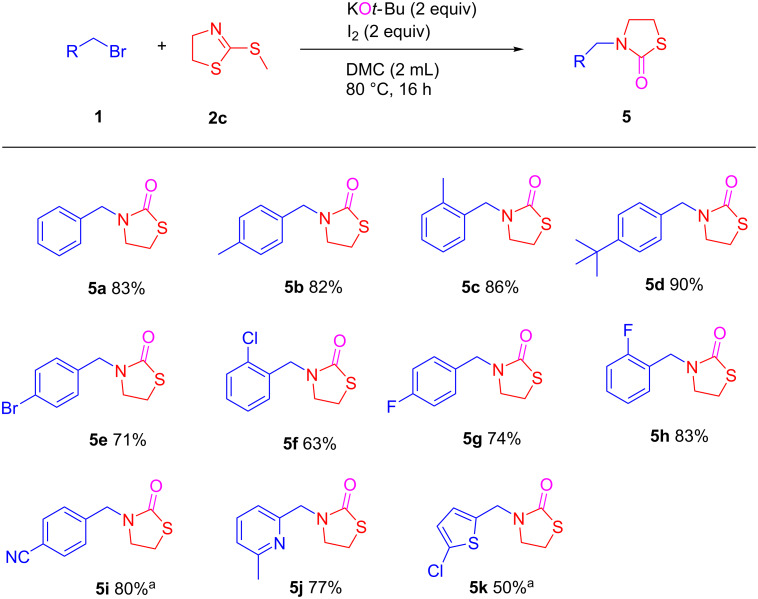
KO*t*-Bu/I_2_-promoted selective *N*-alkylation to synthesis of thiazolidone derivatives. Conditions: KO*t*-Bu (1.0 mmol), 2-(methylthio)-4,5-dihydrothiazole (0.5 mmol), benzyl bromides (1.0 mmol), I_2_ (1.0 mmol) in DMC (2 mL), at 80 °C, under air for 16 h. ^a^With chloromethyl derivative.

Next, the reaction of benzyl bromide (**1a**) with 2-(methylthio)-4,5-dihydrothiazole was performed under similar conditions but with 2 equiv of KO*t*-Bu and 2 equiv of I_2_ at 80 °C (Scheme 5). However, in this case the *N*-substituted thiazolidone compound **5a** was observed as the only product instead of the above 2-aminoethyl acetate compound. Analogously, the *N*-substituted thiazolidone derivatives **5a**–**h** were obtained in 63–90% yields from the corresponding benzyl bromides. The electron-donating and the electron-withdrawing groups did not affect these *N*-alkylation reactions. Interestingly, the reaction tolerates a cyano functional group on the aryl ring of benzyl chloride, and the corresponding thiazolidone **5i** (80%), was directly obtained without reaction of C≡N bond. Substrates with pyridyl and thiophene groups were also applied to the synthesis of the corresponding thiazolidine derivatives **5j** and **5k** in 77% and 50% yields, respectively.

**Scheme 6:**
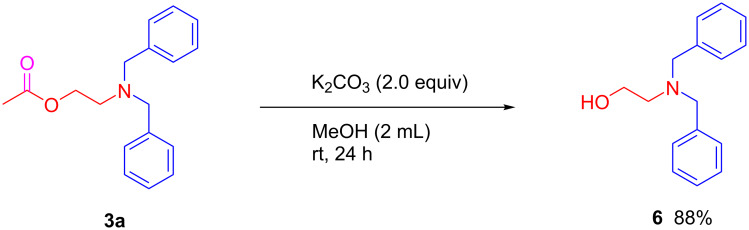
Transformation of 2-aminoethyl acetate derivative to 2-(dibenzylamino)ethanol.

On the other hand, further transformation of the 2-aminoethyl acetate product **3a** was investigated, and 88% yield of 2-(dibenzylamino)ethanol (**6**) was successfully produced in the presence of 2.0 equiv of K_2_CO_3_ in MeOH at room temperature for 24 h (Scheme 6). This result indicated that these types of 2-aminoethyl acetate products are useful building blocks for functionalized alcohols.

**Scheme 7:**
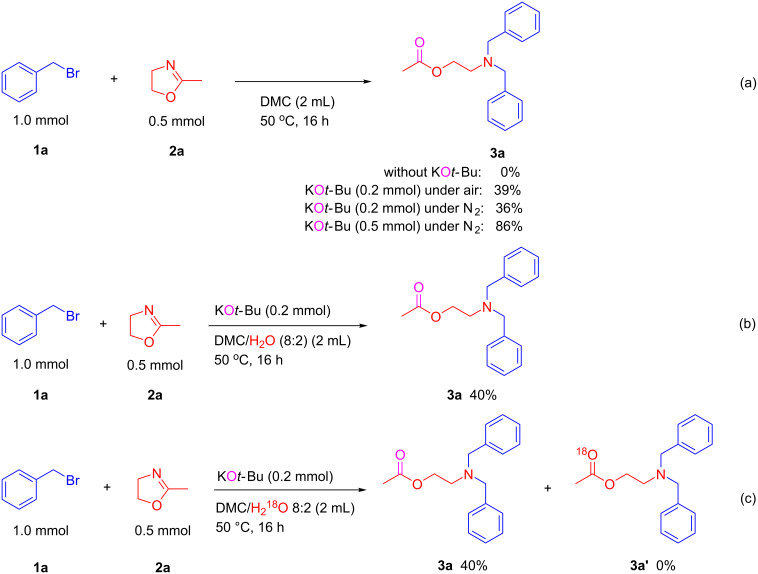
Control experiments and ^18^O-labeling experiment.

Next, to gather more information about this reaction, some control experiments were performed under the established ring-opening *N*-alkylation conditions. First, no conversion to the desired product **3a** was observed without the addition of KO*t*-Bu. However, the addition of 0.2 equiv of KO*t*-Bu gave the 2-aminoethyl acetate product **3a** in 39% yield under air and 36% yield under a N_2_ atmosphere, whereas 86% yield of **3a** were obtained in the presence of 1.0 equiv of KO*t*-Bu under N_2_ conditions (Scheme 7a). These results indicate that KO*t*-Bu plays an important role to improve the yield of 2-aminoethyl acetate product from this ring-opening *N*-alkylation. Then, when this reaction is performed in a mixed solvent system (DMC/H_2_O 8:2), only 40% yield of the desired product **3a** was produced, while no labeled compound was detected by GC–MS in the ^18^O-labeled experiment (Scheme 7b and c). These important results revealed that the oxygen of product **3a** does not come from water or air, and it may be transferred from KO*t*-Bu as supported by Dash and co-worker who demonstrated KO*t*-Bu can serve as an oxygen source [26]. Therefore, in the present KO*t*-Bu-promoted ring-opening *N*-alkylation, KO*t*-Bu not only plays an important role to promote this type of reaction, but also acts as a nucleophilic oxygen donor during the C=N bond cleavage process to lead the corresponding 2-aminoethyl acetates.

**Scheme 8:**
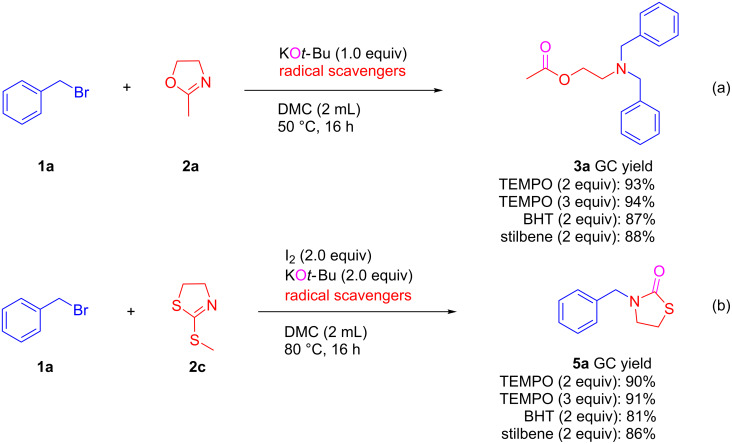
Control experiments with radical scavengers.

To gain insight into the reaction mechanism, the reaction was repeated in the presence of radical scavengers to evaluate if a radical process is involved in the reaction. Excellent yields of products **3a** or **5a** were obtained in the presence of the radical scavengers (2,2,6,6-tetramethylpiperidin-1-yl)oxyl (TEMPO), stilbene, or butylated hydroxytoluene (BHT) in the reaction of benzyl bromide with 2-methyl-2-oxazoline or 2-(methylthio)-4,5-dihydrothiazole (Scheme 8). These experimental results suggest that the reaction may proceed through a nucleophilic substitution rather than a radical pathway.

**Scheme 9:**
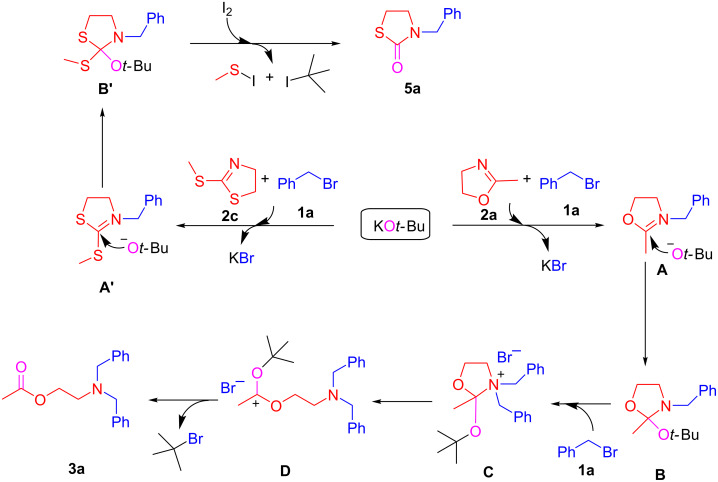
Proposed mechanism.

On the basis of the results collected from the control experiments and previous works in the literature, a plausible mechanism for this KO*t*-Bu-promoted ring-opening *N*-alkylation is illustrated in Scheme 9. The reaction is proposed to begin with the generation of iminium ether **A** [27–28], generated from the reaction of 2-metyl-2-oxazoline with benzyl bromide in the presence of KO*t*-Bu with release KBr. A subsequent second nucleophilic substitution of nitrogen to benzyl bromide would form an ammonium intermediate **C**. The final product **3a** would be produced after rearrangement and release of *tert*-butyl bromide. On the other hand, added I_2_ would react with intermediate **B’** and lead to the thiazolidone compound **5a**.

## Conclusion

In summary, we have developed a new and simple transition-metal-free selective ring-opening *N*-alkylation of 2-methyl-2-oxazoline or 2-(methylthio)-4,5-dihydrothiazole with benzyl halides and allyl halides under mild conditions. Various 2-aminoethyl acetates and *N*-substituted thiazolidinone derivatives were successfully isolated in moderate to excellent yields. Moreover, in this reaction system, KO*t*-Bu not only plays an important role to promote this ring-opening *N*-alkylation, but also acts as an oxygen donor.

## Experimental

### General procedure for the KO*t*-Bu-catalyzed ring-opening *N*-alkylation of 2-oxazolines with benzyl bromides

KO*t*-Bu (0.5 mmol, 56 mg), 2-oxazoline (0.5 mmol), benzyl bromide (1.0 mmol) and DMC (2 mL) were introduced in a tube, equipped with magnetic stirring bar and the mixture was stirred at 50 °C. After 16 h, the progress of the reaction was analyzed by gas chromatography. The solvent was then evaporated under vacuum and the desired product was purified by silica gel chromatography and a mixture of petroleum ether/ethyl acetate as eluent.

### General procedure for the KO*t*-Bu-catalyzed ring-opening *N*-alkylation of 2-oxazolines with benzyl chlorides

KO*t*-Bu (0.5 mmol, 56 mg), I_2_ (0.5 mmol, 127 mg), 2-oxazoline (0.5 mmol), benzyl chloride (1.0 mmol) and DMC (2 mL) were introduced in a tube, equipped with magnetic stirring bar and the mixture was stirred at 80 °C. After 16 h, the progress of the reaction was analyzed by gas chromatography. The solvent was then evaporated under vacuum and the desired product was purified by silica gel chromatography and a mixture of petroleum ether/ethyl acetate as eluent.

### General procedure for KO*t*-Bu/I_2_-promoted *N*-alkylation of thiazolidin-2-one derivatives

KO*t*-Bu (1 mmol, 112 mg), I_2_ (1 mmol, 254 mg), 2-(methylthio)-4,5-dihydrothiazole (0.5 mmol), benzyl halide (1.0 mmol) and DMC (2 mL) were introduced in a tube, equipped with magnetic stirring bar and the mixture was stirred at 80 °C. After 16 h, the progress of the reaction was analyzed by gas chromatography. The solvent was then evaporated under vacuum and the desired product was purified by silica gel chromatography and a mixture of petroleum ether/ethyl acetate as eluent.

## Supporting Information

File 1 Characterization data and copies of NMR spectra.
