# Supplementary material for: KOt-Bu-promoted selective ring-opening N-alkylation of 2-oxazolines to access 2-aminoethyl acetates and N-substituted thiazolidinones
Source: Beilstein J Org Chem. 2020 Mar 25;16:492–501. doi: 10.3762/bjoc.16.44 (PMC7113552; doi:10.3762/bjoc.16.44)

BEILSTEIN JOURNAL OF ORGANIC CHEMISTRY

Supporting Information

for

KO*t*-Bu-promoted selective ring-opening *N*-alkylation of 2-oxazolines to access 2-aminoethyl acetates and *N*-substituted thiazolidinones

Qiao Lin, Shiling Zhang and Bin Li

Beilstein J. Org. Chem. **2020,** *16,* 492–501. doi:10.3762/bjoc.16.44

Characterization data and copies of NMR spectra

License and Terms: This is a supporting information file under the terms of the Creative Commons Attribution License (http://creativecommons.org/licenses/by/4.0). Please note that the reuse, redistribution and reproduction in particular requires that the authors and source are credited.
The license is subject to the *Beilstein Journal of Organic Chemistry* terms and conditions: (https://www.beilstein-journals.org/bjoc)

Table of Contents

General remarks S2

Synthetic procedures S2

Characterization data of products S4

^{1}H and ^{13}C NMR spectra S11

General remarks

All reagents were obtained from commercial sources and were used as received. Technical grade petroleum ether (bp 40–60 °C) and ethyl acetate were used for column chromatography. ^{1}H NMR spectra were recorded in $CDCl_3$ at ambient temperature on Bruker AVANCE I 300 or 400 spectrometers at 300.1 or 400.1 MHz, using the residual solvent signal as internal standard (7.26 ppm). ^{13}C NMR spectra were recorded at 75 or 100 MHz and referenced to the internal solvent signals (central peak is 77.2 ppm). Chemical shifts (δ) and coupling constants (J) are given in ppm and in Hz, respectively. The peak patterns are indicated as follows: s, singlet; d, doublet; t, triplet; q, quartet; m, multiplet, and br. for broad. GC analyses were performed with GC-14C (Shimadzu) equipped with a 30 m capillary column (Supelco, SPB-5, fused silica capillary column, 30 M × 0.25 mm × 0.25 mm film thickness), was used with N_2/air as vector gas. GC–MS were measured by GCMS-7890A-5975C (Agilent) with GC-7890A equipped with a 30 m capillary column (HP-5ms, fused silica capillary column, 30 M*0.25 mm*0.25 mm film thickness), was used with helium as vector gas. HRMS were measured by MAT 95XP (Termol) (LCMS-IT-TOF). The following GC conditions were used: initial temperature 80 °C, for 2 minutes, then rate 20 °C/min until 260 °C and 260 °C for 20 minutes.

Gram scale procedure for synthesis of 2-(dibenzylamino)ethyl acetate (3a)

KO*t*-Bu (10 mmol, 1.12 g), 2-methyl-2-oxazole (10 mmol, 0.85 mL), benzyl bromide (20 mmol, 2.38 mL) and DMC (10 mL) were introduced in a tube, equipped with a magnetic stirring bar and the mixture was stirred at 50 ° C. After 16 h, the conversion of the reaction was analyzed by gas chromatography. The solvent was then evaporated under vacuum and the desired product was purified by silica gel chromatography using a mixture of petroleum ether/ethyl acetate as eluent to afford 2.38 g (84%) of **3a** as light yellow oil.

Procedure for the synthesis of 2-(dibenzylamino)ethanol (6)

K_2CO_3 (1.0 mmol, 112 mg), 2-(dibenzylamino)ethyl acetate (0.5 mmol, 142 μL), and methanol (2 mL) were introduced in a tube, equipped with a magnetic stirring bar and the mixture was stirred at room temperature. After 24 h, the conversion of the reaction was analyzed by gas chromatography. The solvent was then evaporated under vacuum and the desired product was purified by silica gel column chromatography using a mixture of petroleum ether/ethyl acetate as eluent to afford 106 mg (88%) of product **6** as light yellow oil.

Table S1: Optimization of the KO*t*-Bu-promoted selective ring-opening *N*-alkylation of 2-(methylthio)-4,5-dihydrothiazole with benzyl bromide.[a]

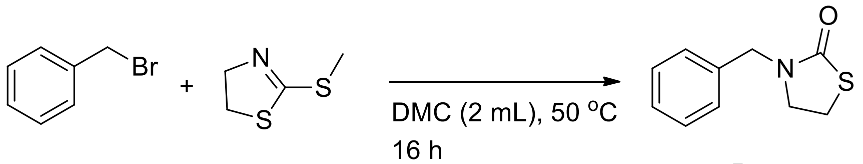

Entry	Base (equiv.)	Additive (equiv.)	GC-yield (%)
1	KO^{t}Bu (1)	---	---
2	KO^{t}Bu (2)	---	---
3	KO^{t}Bu (1)	CuBr (0.2)	---
4	KO^{t}Bu (1)	$CuBr_2$ (0.2)	---
5	KO^{t}Bu (1)	I_2 (1)	48
6	KO^{t}Bu (2)	I_2 (1)	73
5	KO^{t}Bu (2)	I_2 (2)	99

[a]KO*t*-Bu, additive, 2-(methylthio)-4,5-dihydrothiazole (0.5 mmol), benzyl bromide (1.0 mmol), DMC (2 mL), under air for 16 h.

Characterization data of products

2-(Dibenzylamino)ethyl acetate (3a)

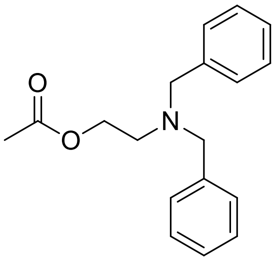

Light yellow oil, yield = 80%, 113 mg, ^{1}H NMR (300 MHz, $CDCl_3$): δ = 7.43-7.28 (m, 10H), 4.21 (t, 2H, *J* = 6.0 Hz), 3.69 (s, 4H), 2.77 (t, 2H, *J* = 6.0 Hz), 2.07 (s, 3H). $^{13}C\{^1H\}$ NMR (75 MHz, $CDCl_3$): δ = 171.1, 139.5, 128.9, 128.4, 127.1, 62.5, 58.8, 51.8, 21.1. HRMS (EI): *m/z* calcd for $C_{18}H_{22}NO_2$ $[M+H]^+$ 284.1645, found 284.1640.

2-(Bis(4-methylbenzyl)amino)ethyl acetate (3b)

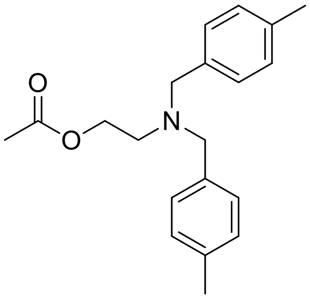

Organge oil, yield = 85%, 132 mg, ^{1}H NMR (300 MHz, $CDCl_3$): δ = 7.32 (d, 4H, *J* = 8.1 Hz), 7.18 (d, 4H, *J* = 7.8 Hz), 4.22 (t, 2H, *J* = 6.0 Hz), 3.67 (s, 4H), 2.77 (t, 2H, *J* = 6.0 Hz), 2.40 (s, 6H), 2.09 (s, 3H). $^{13}C\{^1H\}$ NMR (75 MHz, $CDCl_3$): δ = 171.1, 136.6, 136.4, 129.0, 128.8, 62.6, 58.4, 51.5, 21.2, 21.1. HRMS (EI): *m/z* calcd for $C_{20}H_{26}NO_2$ $[M+H]^+$ 312.1958, found 312.1966.

2-(Bis(2-methylbenzyl)amino)ethyl acetate (3c)

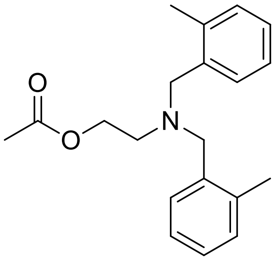

Light yellow oil, yield = 73%, 113 mg, ^{1}H NMR (300 MHz, $CDCl_3$): δ = 7.40-7.37 (m, 2H), 7.22-7.18 (m, 6H), 4.18 (t, 2H, *J* = 6.0 Hz), 3.67 (s, 4H), 2.77 (t, 2H, *J* = 5.7 Hz), 2.34 (s, 6H), 2.05 (s, 3H). $^{13}C\{^1H\}$ NMR (75 MHz, $CDCl_3$): δ = 171.0, 137.5, 137.1, 130.4, 130.2, 127.2, 125.6, 62.5, 57.6, 52.3, 21.1, 19.2. HRMS (EI): *m/z* calcd for $C_{20}H_{26}NO_2$ $[M+H]^+$ 312.1958, found 312.1952.

2-(Bis(4-(*tert*-butyl)benzyl)amino)ethyl acetate (3d)

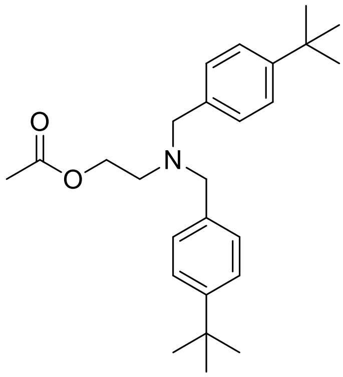

Light yellow oil, yield = 78%, 154 mg, ^{1}H NMR (300 MHz, $CDCl_3$): δ = 7.42-7.36 (m, 8H), 4.26 (t, 2H, *J* = 6.0 Hz), 3.70 (s, 4H), 2.80 (t, 2H, *J* = 6.0 Hz), 2.10 (s, 3H), 1.39 (s, 18H). ^{13}C{^{1}H} NMR (75 MHz, $CDCl_3$): δ = 171.1, 149.9, 136.5, 128.5, 125.2, 62.7, 58.3, 51.8, 34.6, 31.6, 21.1. HRMS (EI): *m/z* calcd for $C_{26}H_{38}NO_2$ $[M+H]^+$ 396.2897, found 396.2902.

2-(Bis(4-fluorobenzyl)amino)ethyl acetate (3e)

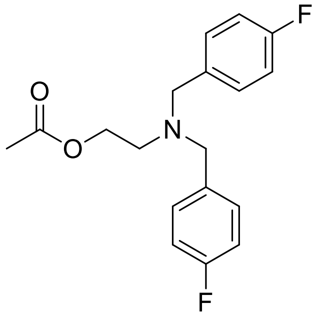

Brown oil, yield = 79%, 126 mg, ^{1}H NMR (300 MHz, $CDCl_3$): δ = 7.35-7.30 (m, 4H), 7.05-6.99 (m, 4H), 4.17 (t, 2H, *J* = 5.7 Hz), 3.60 (s, 4H), 2.72 (t, 2H, *J* = 5.7 Hz), 2.05 (s, 3H). ^{13}C{^{1}H} NMR (75 MHz, $CDCl_3$): δ = 171.1, 160.5 (d, J_{CF} = 243.3 H_Z), 135.0 (d, J_{CF} = 3.075 H_Z), 130.2 (d, J_{CF} = 7.875 H_Z), 115.1 (d, J_{CF} = 21.075 H_Z), 62.3, 57.9, 51.7, 21.1. HRMS (EI): *m/z* calcd for $C_{18}H_{20}F_2NO_2$ $[M+H]^+$ 320.1457, found 320.1451.

2-(bis(2-fluorobenzyl)amino)ethyl acetate (3f)

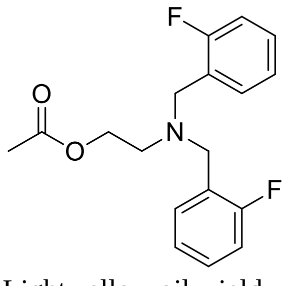

Light yellow oil, yield = 72%, 115 mg, ^{1}H NMR (300 MHz, $CDCl_3$): δ = 7.53-7.48 (m, 2H), 7.29-7.01 (m, 6H), 4.22 (t, 2H, *J* = 6.0 Hz), 3.78 (s, 4H), 2.79 (t, 2H, *J* = 6.0 Hz), 2.05 (s, 3H). ^{13}C{^{1}H} NMR (75 MHz, $CDCl_3$): δ = 171.1, 159.8 (d, J_{CF} = 244.4 H_Z), 131.1 (d, J_{CF} = 4.5 H_Z), 128.7 (d, J_{CF} = 8.175 H_Z), 125.8 (d, J_{CF} = 13.875 H_Z), 124.0 (d, J_{CF} = 3.6 H_Z), 115.2 (d, J_{CF} = 22.05 H_Z), 62.4, 51.9, 51.3 (d, J_{CF} = 2.25 H_Z), 21.0. HRMS (EI): *m/z* calcd for $C_{18}H_{19}F_2NO_2Na$ $[M+Na]^+$ 342.1276, found 342.1279.

2-(Bis(2-chlorobenzyl)amino)ethyl acetate (3g)

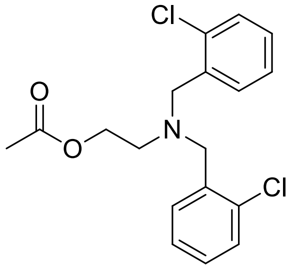

Light yellow oil, yield = 70%, 123 mg, ^{1}H NMR (300 MHz, $CDCl_3$): δ = 7.62-7.59 (m, 2H), 7.37-7.16 (m, 6H), 4.24 (t, 2H, *J* = 6.0 Hz), 3.86 (s, 4H), 2.84 (t, 2H, *J* = 6.0 Hz), 2.06 (s, 3H). $^{13}C\{^1H\}$ NMR (75 MHz, $CDCl_3$): δ = 171.0, 136.8, 134.1, 130.5, 129.5, 128.2, 126.8, 62.5, 55.9, 52.6, 21.1. HRMS (EI): *m/z* calcd for $C_{18}H_{20}Cl_2NO_2$ $[M+H]^+$ 352.0866, found 352.0861.

2-(Diallylamino)ethyl acetate (3h)

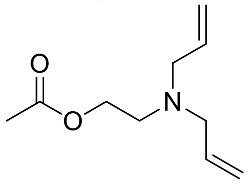

Light yellow oil, yield = 69%, 63 mg, ^{1}H NMR (300 MHz, $CDCl_3$): δ = 5.95-5.81 (m, 2H), 5.26-5.19 (m, 4H), 4.19 (t, 2H, *J* = 6.0 Hz), 3.22 (d, 4H, *J* = 6.3 Hz), 2.79 (t, 2H, *J* = 6.0 Hz), 2.07 (s, 3H). $^{13}C\{^1H\}$ NMR (75 MHz, $CDCl_3$): δ = 171.1, 134.3, 119.0, 62.1, 57.3, 51.2, 21.2. HRMS (EI): *m/z* calcd for $C_{10}H_{18}NO_2$ $[M+H]^+$ 184.1332, found 184.1339.

(2*E*,2'*E*)-Dimethyl 4,4'-((2-acetoxyethyl)azanediyl)bis(but-2-enoate) (3i)

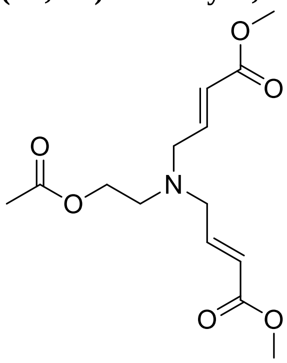

Light red oil, yield = 71%, 106 mg, ^{1}H NMR (300 MHz, $CDCl_3$): δ = 6.92-6.83 (m, 2H), 6.04-5.98 (m, 2H), 4.10 (t, 2H, *J* = 5.7 Hz), 3.71 (s, 6H), 3.27 (d, 4H, *J* = 5.7 Hz), 2.70 (t, 2H, *J* = 5.7 Hz), 2.05 (s, 3H). $^{13}C\{^1H\}$ NMR (75 MHz, $CDCl_3$): δ = 171.0, 166.6, 145.4, 122.9, 62.1, 55.3, 52.5, 51.6, 21.0. HRMS (EI): *m/z* calcd for $C_{14}H_{22}NO_6$ $[M+H]^+$ 300.1442, found 300.1448.

(2*E*,2'*E*)-Diethyl 4,4'-((2-acetoxyethyl)azanediyl)bis(but-2-enoate) (3j)

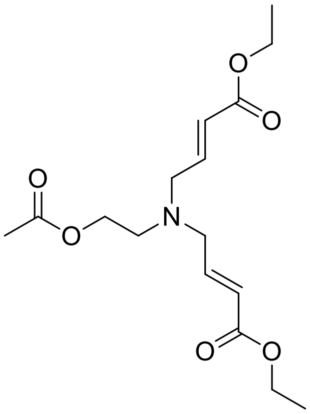

Brown oil, yield = 58%, 95 mg, ^{1}H NMR (300 MHz, $CDCl_3$): δ = 6.93-6.85 (m, 2H), 6.01 (d, 2H, *J* = 15.6 Hz), 4.22-4.10 (m, 6H), 3.28 (d, 4H, *J* = 5.4 Hz), 2.74-2.70 (m, 2H), 2.07 (s, 3H), 1.31-1.25 (m, 6H). $^{13}C\{^{1}H\}$ NMR (75 MHz, $CDCl_3$): δ = 171.0, 166.2, 145.1, 123.3, 62.2, 60.5, 55.3, 52.5, 21.0, 14.3. HRMS (EI): *m/z* calcd for $C_{16}H_{26}NO_6$ $[M+H]^+$ 328.1755, found 328.1757.

2-(Bis((5-chlorothiophen-2-yl)methyl)amino)ethyl acetate (3k)

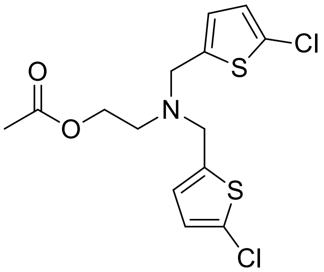

Brown oil, yield = 66%, 120 mg, ^{1}H NMR (300 MHz, $CDCl_3$): δ = 6.75-6.69 (m, 4H), 4.20 (t, 2H, *J* = 6.0 Hz), 3.81 (s, 4H), 2.80 (t, 2H, *J* = 6.0 Hz), 2.10 (s, 3H). $^{13}C\{^{1}H\}$ NMR (75 MHz, $CDCl_3$): δ = 171.0, 141.5, 129.5, 125.6, 125.1, 62.1, 53.1, 51.2, 21.1. HRMS (EI): *m/z* calcd for $C_{14}H_{16}Cl_2NO_2S_2$ $[M+H]^+$ 363.9994, found 363.9997.

2-(Bis(4-methylbenzyl)amino)-2-methylpropyl acetate (3l)

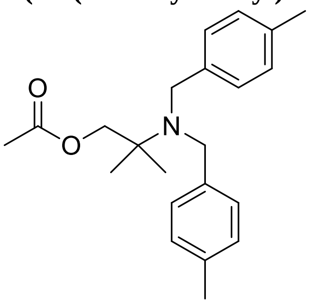

Yellow oil, yield = 72%, 122 mg, ^{1}H NMR (300 MHz, $CDCl_3$): δ = 7.21 (d, 4H, *J* = 7.8 Hz), 7.06 (d, 4H, *J* = 7.8 Hz), 4.11 (s, 2H), 3.79 (s, 4H), 2.31 (s, 6H), 2.11 (s, 3H), 1.19 (s, 6H). $^{13}C\{^{1}H\}$ NMR (75 MHz, $CDCl_3$): δ = 171.2, 139.2, 135.9, 128.7, 128.3, 70.1, 57.8, 53.6, 23.3, 21.2, 21.1. HRMS (EI): *m/z* calcd for $C_{22}H_{30}NO_2$ $[M+H]^+$ 340.2271, found 340.2274.

3-Benzylthiazolidin-2-one[1,2] (5a)

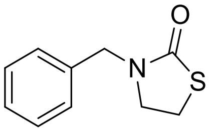

Light yellow oil, yield = 83%, 80 mg, ^{1}H NMR (400 MHz, $CDCl_3$): δ = 7.37-7.26 (m, 5H), 4.48 (s, 2H), 3.51 (t, 2H, *J* = 7.2 Hz), 3.22 (t, 2H, *J* = 7.2 Hz). $^{13}C\{^{1}H\}$ NMR (100 MHz, $CDCl_3$): δ =

172.2, 136.0, 128.8, 128.1, 127.9, 48.6, 48.0, 25.5. HRMS (EI): *m/z* calcd for $C_{10}H_{12}ONS$ $[M+H]^+$ 194.0634, found 194.0639.

3-(4-Methylbenzyl)thiazolidin-2-one[2] (5b)

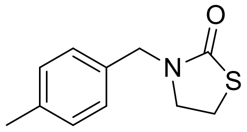

Yellow oil, yield = 82%, 85 mg, ^1H NMR (400 MHz, $CDCl_3$): δ = 7.28-7.17 (m, 4H), 4.45 (s, 2H), 3.51 (t, 2H, *J* = 7.2 Hz), 3.22 (t, 2H, *J* = 7.6 Hz), 2.36 (s, 3H). $^{13}C\{^1H\}$ NMR (100 MHz, $CDCl_3$): δ = 172.2, 137.7, 133.0, 129.5, 128.3, 128.2, 48.5, 48.0, 25.6, 21.2. HRMS (EI): *m/z* calcd for $C_{11}H_{14}ONS$ $[M+H]^+$ 208.0791, found 208.0796.

3-(2-Methylbenzyl)thiazolidin-2-one[2] (5c)

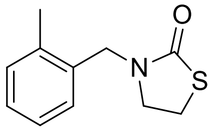

Colorless solid, yield = 86%, 89 mg, ^1H NMR (600 MHz, $CDCl_3$): δ = 7.22-7.17 (m, 4H), 4.49 (s, 2H), 3.44 (t, 2H, *J* = 7.2 Hz), 3.21 (t, 2H, *J* = 7.2 Hz), 2.31 (s, 3H). $^{13}C\{^1H\}$ NMR (150 MHz, $CDCl_3$): δ = 171.9, 136.9, 133.9, 130.8, 129.0, 128.1, 126.3, 48.0, 46.9, 25.6, 19.2. HRMS (EI): *m/z* calcd for $C_{11}H_{14}ONS$ $[M+H]^+$ 208.0791, found 208.0792.

3-(4-(*tert*-Butyl)benzyl)thiazolidin-2-one[2] (5d)

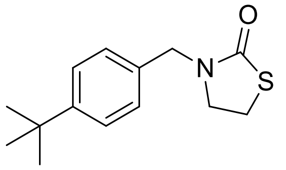

Colorless solid, yield = 90%, 112 mg, ^1H NMR (400 MHz, $CDCl_3$): δ = 7.37 (d, 2H, *J* = 8.0 Hz), 7.21 (d, 2H, *J* = 8.4 Hz), 4.47 (s, 2H), 3.53 (t, 2H, *J* = 7.2 Hz), 3.23 (t, 2H, *J* = 7.6 Hz), 1.33 (s, 9H). $^{13}C\{^1H\}$ NMR (100 MHz, $CDCl_3$): δ = 172.2, 150.9, 133.0, 128.0, 125.8, 48.4, 48.1, 34.6, 31.4, 25.5. HRMS (EI): *m/z* calcd for $C_{14}H_{20}ONS$ $[M+H]^+$ 250.1260, found 250.1262.

3-(4-Bromobenzyl)thiazolidin-2-one (5e)

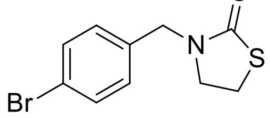

Colorless oil, yield = 71%, 96 mg, ^1H NMR (400 MHz, $CDCl_3$): δ = 7.57 (d, 2H, *J* = 8.4 Hz), 7.15 (d, 2H, *J* = 8.4 Hz), 4.43 (s, 2H), 3.50 (t, 2H, *J* = 7.2 Hz), 3.24 (t, 2H, *J* = 7.2 Hz). $^{13}C\{^1H\}$ NMR (100 MHz, $CDCl_3$): δ = 172.4, 135.2, 132.0, 129.9, 121.9, 48.1, 48.0, 25.6. HRMS (EI): *m/z* calcd for $C_{10}H_{11}ONBrS$ $[M+H]^+$ 271.9739, found 271.9742.

3-(2-Chlorobenzyl)thiazolidin-2-one[2] (5f)

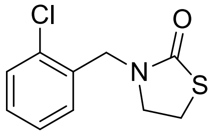

Yellow oil, yield = 63%, 72 mg, ^1H NMR (400 MHz, $CDCl_3$): δ = 7.38-7.24 (m, 4H), 4.62 (s, 2H), 3.57 (t, 2H, *J* = 7.2 Hz), 3.26 (t, 2H, *J* = 7.6 Hz). $^{13}C\{^1H\}$ NMR (100 MHz, $CDCl_3$): δ = 172.4, 133.7, 133.6, 130.0, 129.8, 129.3, 127.4, 48.3, 45.9, 25.7. HRMS (EI): *m/z* calcd for $C_{10}H_{11}ONClS$ $[M+H]^+$ 228.0244, found 228.0246.

3-(4-Fluorobenzyl)thiazolidin-2-one (5g)

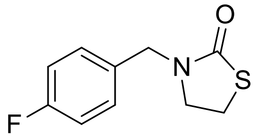

Yellow oil, yield = 74%, 78 mg, ^{1}H NMR (400 MHz, $CDCl_3$): δ = 7.25-7.22 (m, 2H), 7.03-6.99 (m, 2H), 4.43 (s, 2H), 3.49 (t, 2H, J = 7.2 Hz), 3.22 (t, 2H, J = 6.8 Hz). $^{13}C\{^1H\}$ NMR (100 MHz, $CDCl_3$): δ = 172.3, 161.2 (d, J_{CF} = 244.8 H_Z), 131.9 (d, J_{CF} = 3.1 H_Z), 129.9 (d, J_{CF} = 8.1 H_Z), 115.6 (d, J_{CF} = 21.4 H_Z), 48.0, 47.9, 25.5. HRMS (EI): *m/z* calcd for $C_{10}H_{11}ONFS$ $[M+H]^+$ 212.0540, found 212.0538.

3-(2-Fluorobenzyl)thiazolidin-2-one (5h)

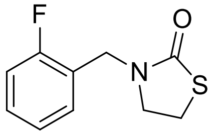

Yellow oil, yield = 83%, 87 mg, ^{1}H NMR (400 MHz, $CDCl_3$): δ = 7.36-7.27 (m, 2H), 7.16-7.04 (m, 2H), 4.55 (s, 2H), 3.58 (t, 2H, J = 7.2 Hz), 3.25 (t, 2H, J = 7.6 Hz). $^{13}C\{^1H\}$ NMR (100 MHz, $CDCl_3$): δ = 172.4, 159.8 (d, J_{CF} = 246.5 H_Z), 130.8 (d, J_{CF} = 3.7 H_Z), 129.8 (d, J_{CF} = 8.1 H_Z), 124.7 (d, J_{CF} = 3.5 H_Z), 122.9 (d, J_{CF} = 15.0 H_Z), 115.5 (d, J_{CF} = 21.6 H_Z), 48.2, 41.9 (d, J_{CF} = 3.9 H_Z), 25.6. HRMS (EI): *m/z* calcd for $C_{10}H_{11}ONFS$ $[M+H]^+$ 212.0540, found 212.0539.

4-((2-Oxothiazolidin-3-yl)methyl)benzonitrile (5i)

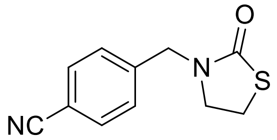

Light yellow solid, yield = 80%, 87 mg, ^{1}H NMR (400 MHz, $CDCl_3$): δ = 7.61 (d, 2H, J = 8.0 Hz), 7.36 (d, 2H, J = 8.0 Hz), 4.51 (s, 2H), 3.52 (t, 2H, J = 7.2 Hz), 3.27 (t, 2H, J = 7.2 Hz). $^{13}C\{^1H\}$ NMR (100 MHz, $CDCl_3$): δ = 172.6, 141.6, 132.6, 128.6, 118.5, 111.7, 48.2, 48.1, 25.5. HRMS (EI): *m/z* calcd for $C_{11}H_{11}ON_2S$ $[M+H]^+$ 219.0587, found 219.0589.

3-((6-Methylpyridin-2-yl)methyl)thiazolidin-2-one (5j)

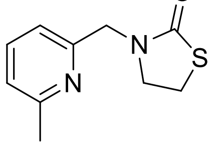

Yellow oil, yield = 77%, 80 mg, ^{1}H NMR (400 MHz, $CDCl_3$): δ = 7.56 (t, 1H, J = 7.6 Hz), 7.10-7.06 (m, 2H), 4.57 (s, 2H), 3.67 (t, 2H, J = 7.2 Hz), 3.27 (t, 2H, J = 7.6 Hz), 2.53 (s, 3H). $^{13}C\{^1H\}$ NMR (100 MHz, $CDCl_3$): δ = 172.5, 158.3, 155.5, 137.4, 122.4, 119.1, 50.5, 48.7, 25.8, 24.5. HRMS (EI): *m/z* calcd for $C_{10}H_{13}ON_2S$ $[M+H]^+$ 209.0743, found 209.0742.

3-((5-Chlorothiophen-2-yl)methyl)thiazolidin-2-one (5k)

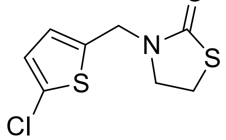

Light yellow solid, yield = 50%, 58 mg, ^{1}H NMR (400 MHz, $CDCl_3$): δ = 6.87-6.62 (m, 2H), 4.53 (s, 2H), 3.59 (t, 2H, J = 7.2 Hz), 3.26 (t, 2H, J = 7.6 Hz). $^{13}C\{^1H\}$ NMR (100 MHz, $CDCl_3$): δ = 172.4, 137.2, 130.3, 126.6, 126.0, 47.9, 43.5, 25.6. HRMS (EI): *m/z* calcd for $C_8H_9ONClS_2$ $[M+H]^+$ 233.9814, found 233.9825.

2-(Dibenzylamino)ethanol[3] (6)

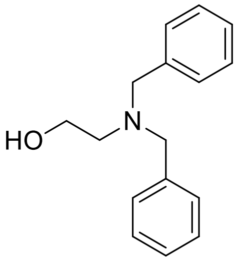

Light yellow oil, yield = 88%, 106 mg, ^{1}H NMR (500 MHz, $CDCl_3$): δ = 7.39-7.29 (m, 10H), 3.67 (s, 4H), 3.62 (t, 2H, J = 5.5 Hz), 2.70 (t, 2H, J = 5.5 Hz), 2.47 (brs, 1H). $^{13}C\{^1H\}$ NMR (125 MHz, $CDCl_3$): δ = 138.9, 129.1, 128.6, 127.4, 58.6, 58.3, 54.8. HRMS (EI): *m/z* calcd for $C_{16}H_{20}NO$ $[M+H]^+$ 242.1539, found 242.1531.

References

1. Mahy, W.; Plucinski, P.; Jover, J.; Frost, C. G. *Angew. Chem. Int. Ed.* **2015**, *54*, 10944.
2. Li, B.; Lin, Q.; Lin, Z.; Zhang, S. *Faming Zhuanli Shenqing*, **2019**, CN 110294719.
3. White, J. D.; Hansen, J. D. *J. Org. Chem.* **2005**, *70*, 1963.

2-(Dibenzylamino)ethyl acetate (3a)

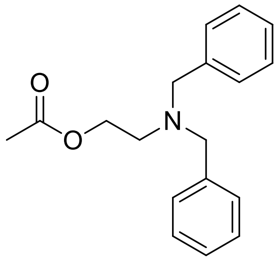

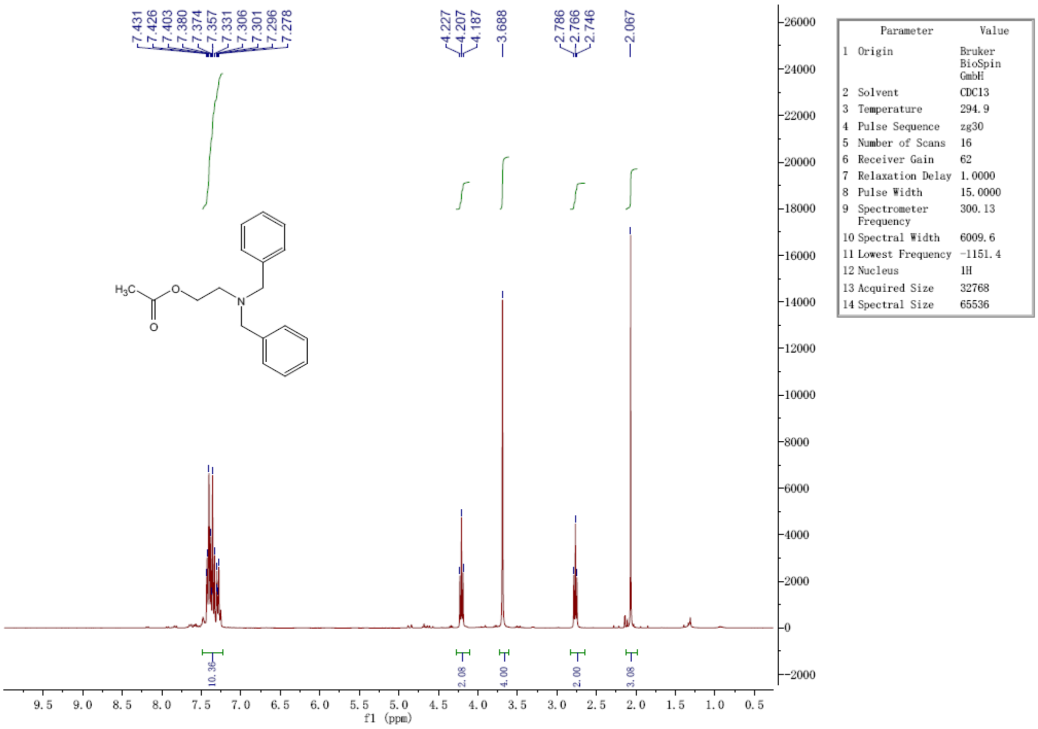

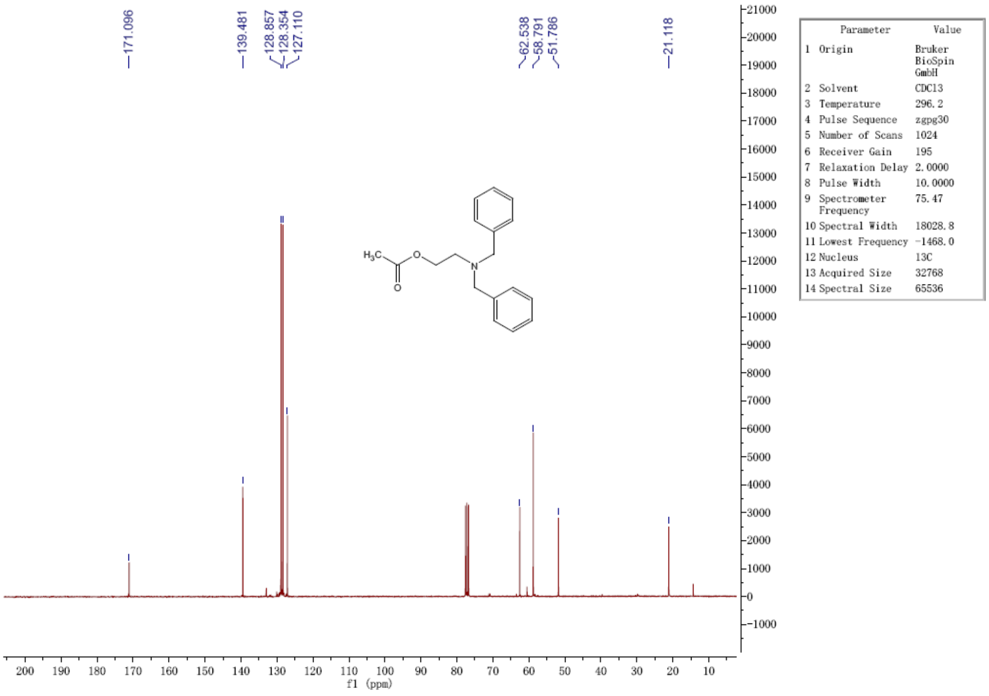

2-(Bis(4-methylbenzyl)amino)ethyl acetate (3b)

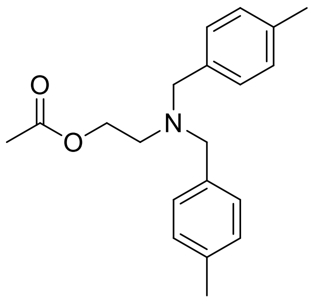

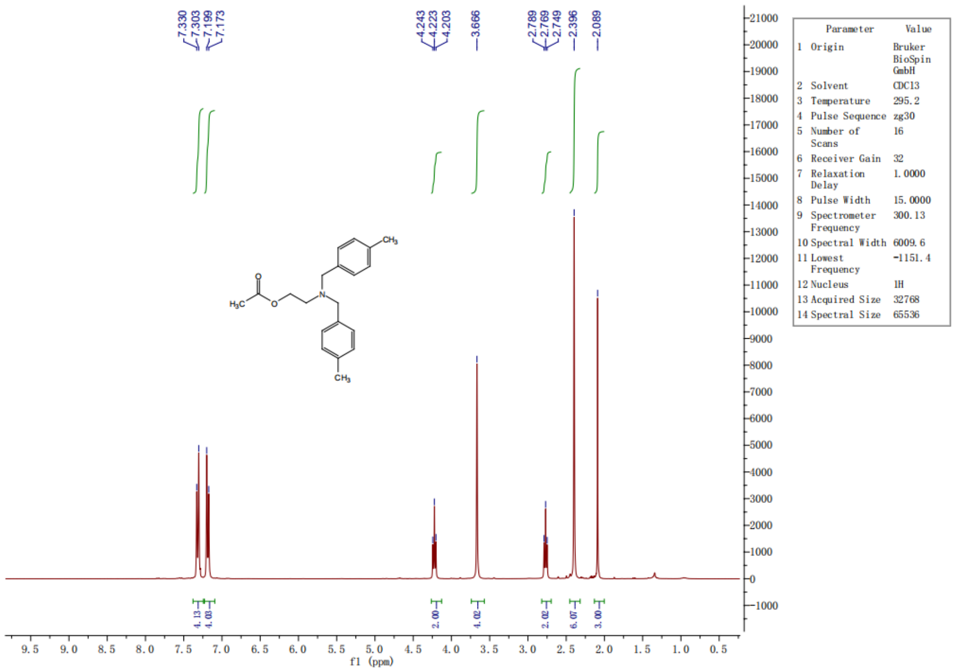

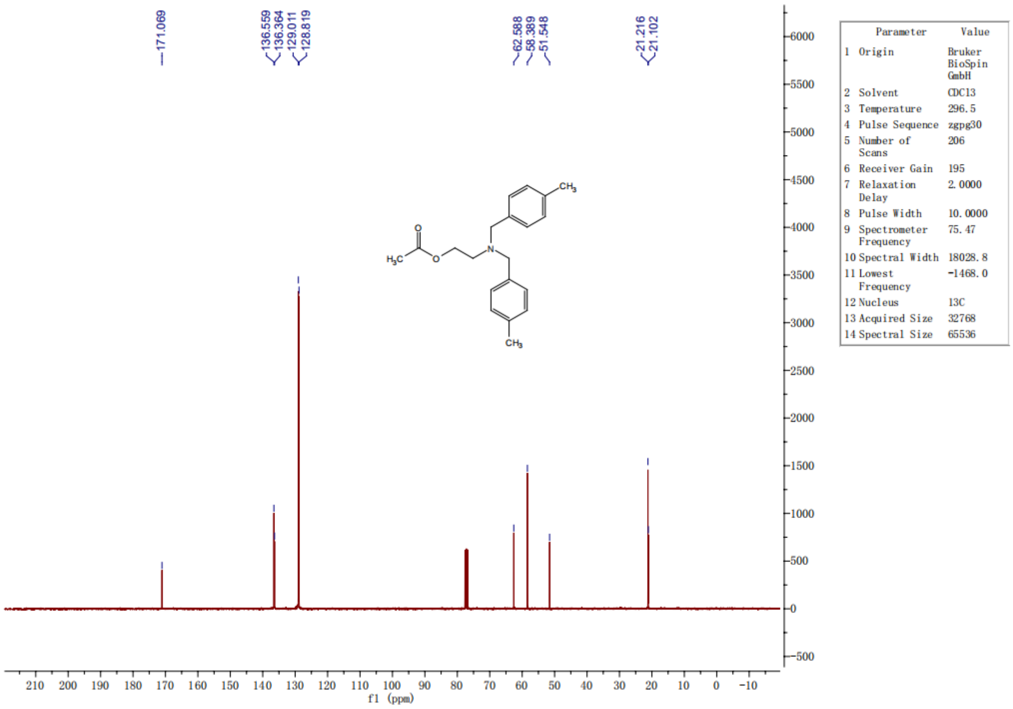

2-(Bis(2-methylbenzyl)amino)ethyl acetate (3c)

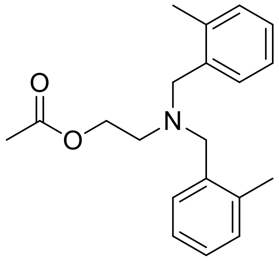

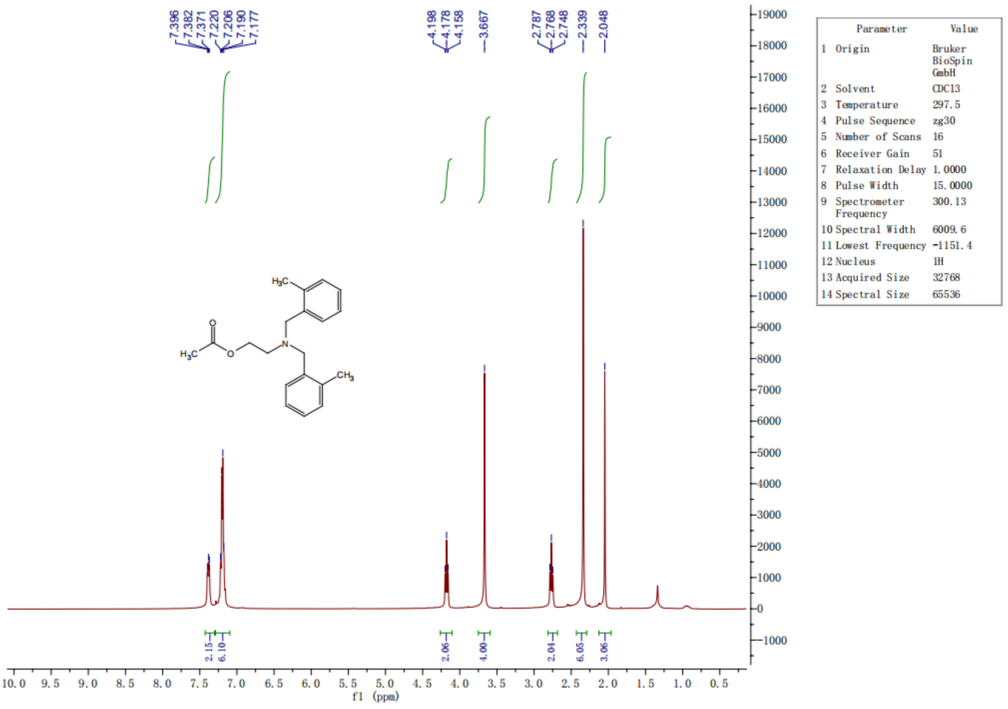

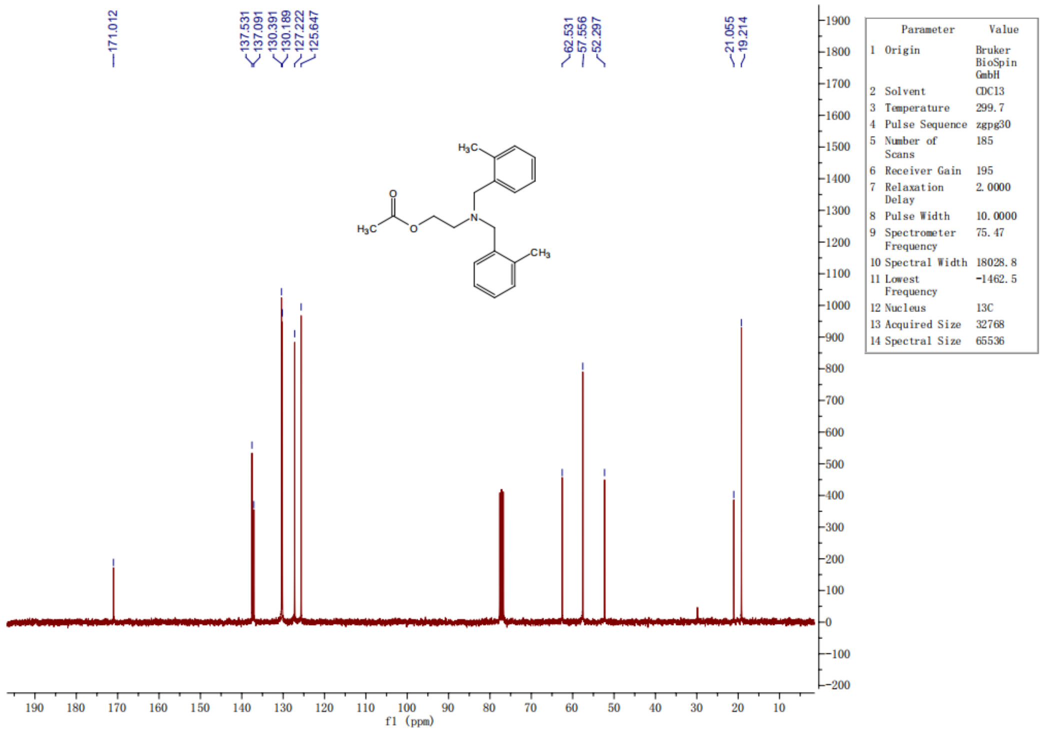

2-(Bis(4-(*tert*-butyl)benzyl)amino)ethyl acetate (3d)

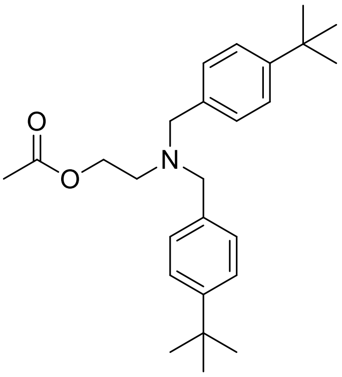

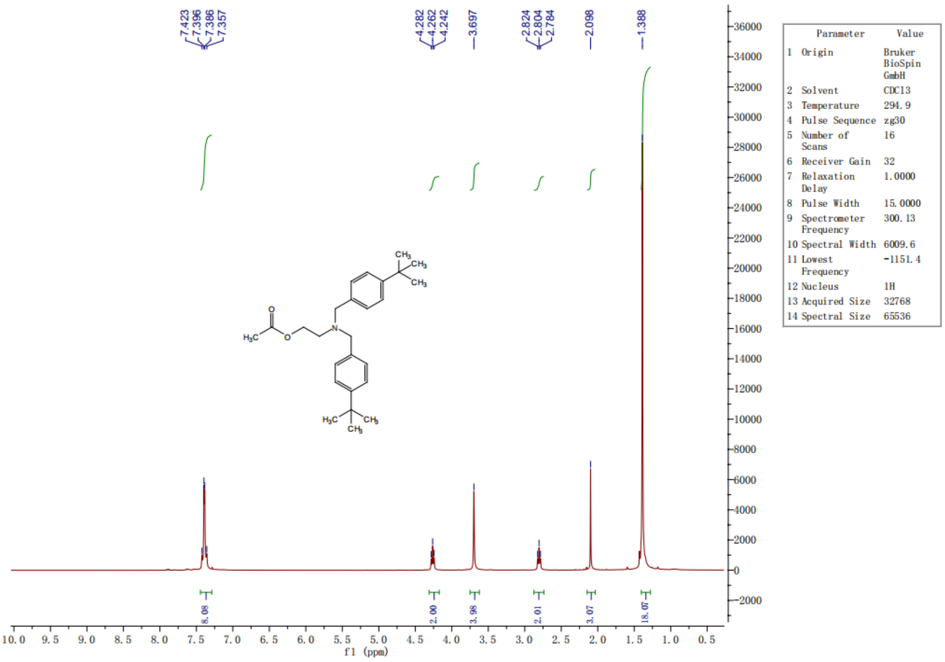

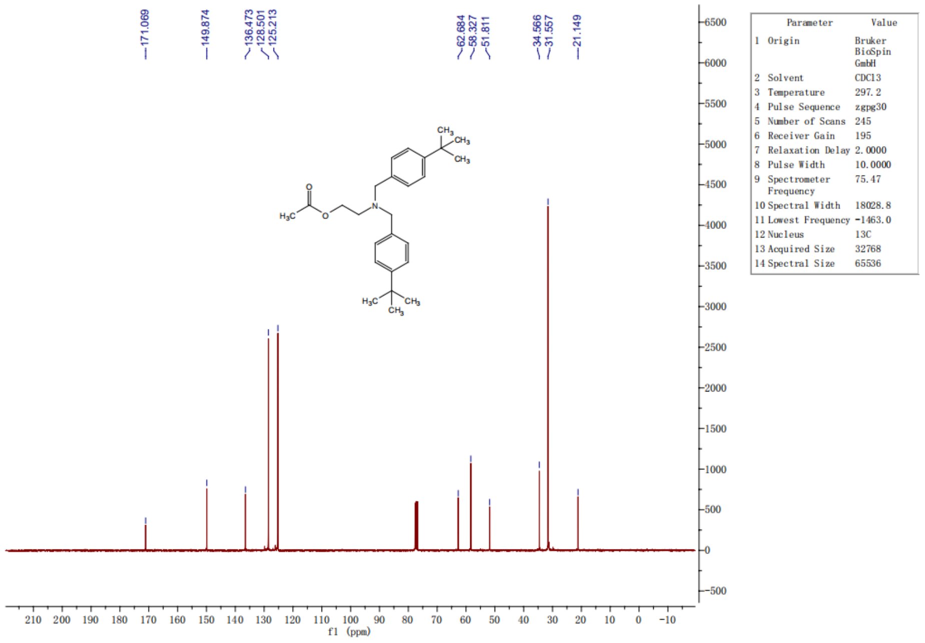

2-(Bis(4-fluorobenzyl)amino)ethyl acetate (3e)

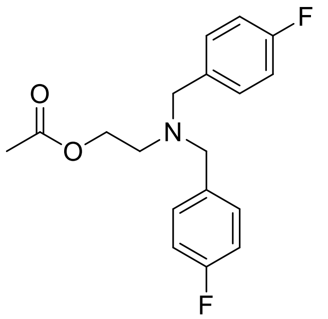

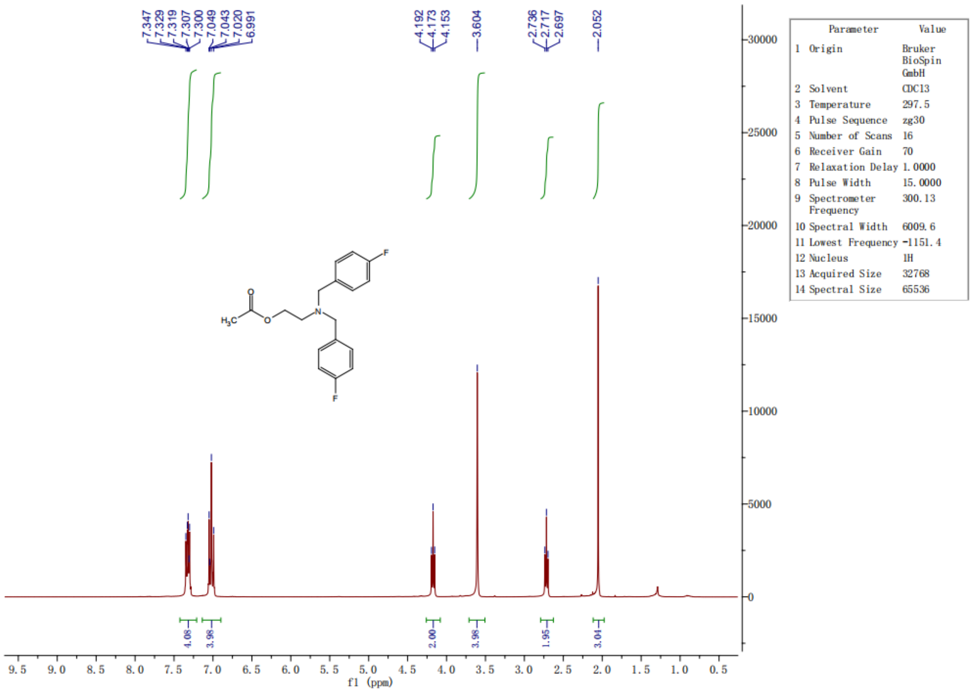

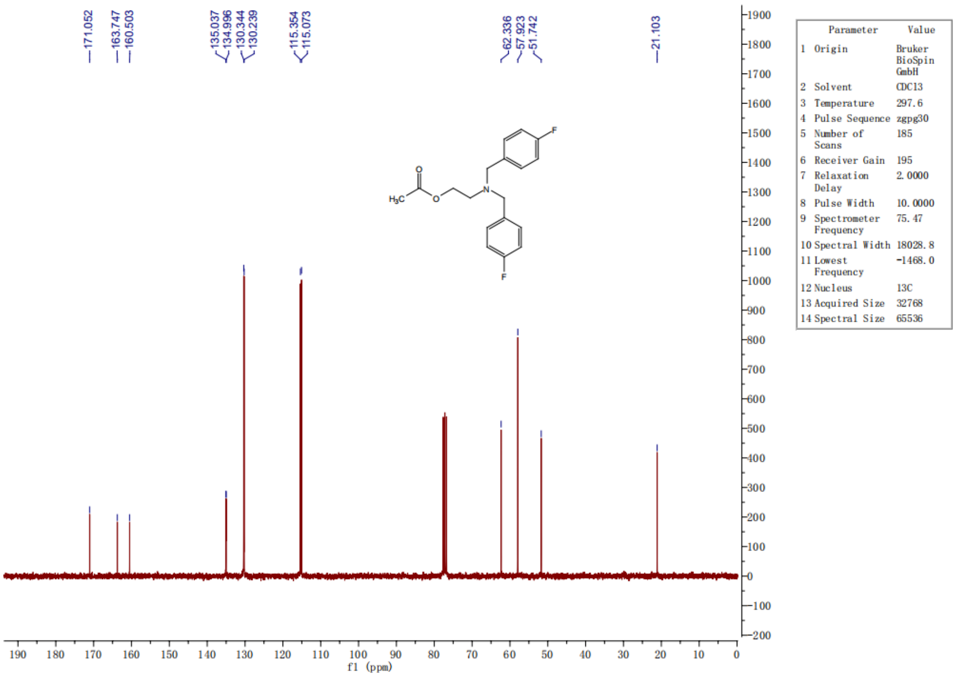

2-(Bis(2-fluorobenzyl)amino)ethyl acetate (3f)

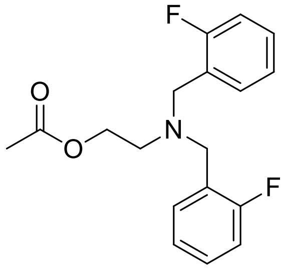

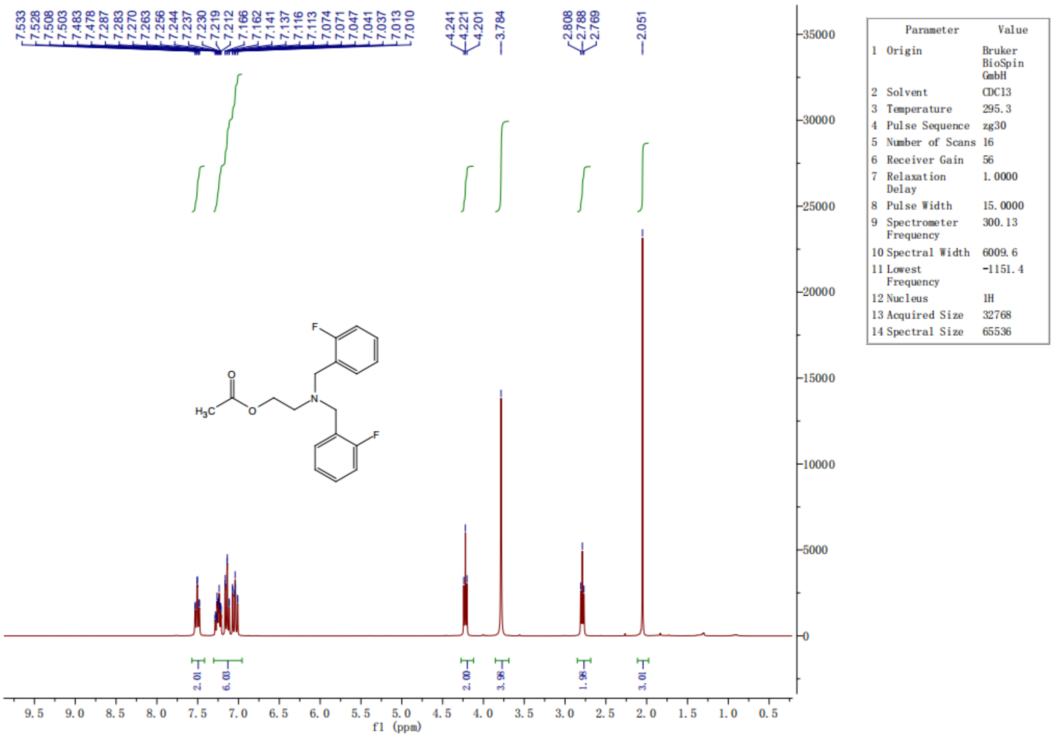

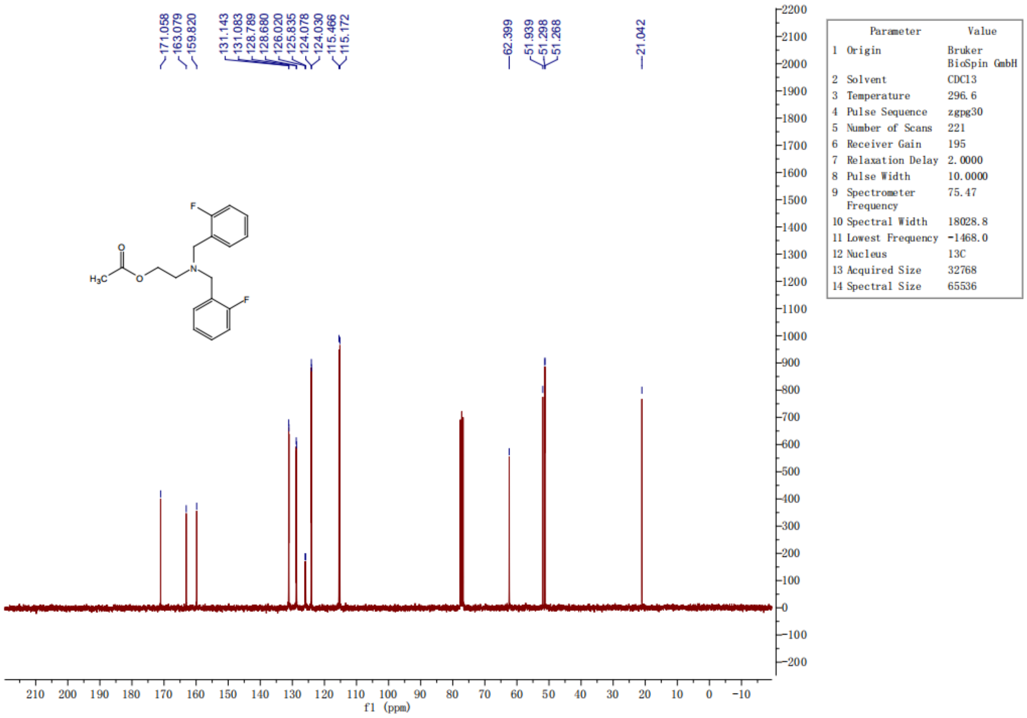

2-(Bis(2-chlorobenzyl)amino)ethyl acetate (3g)

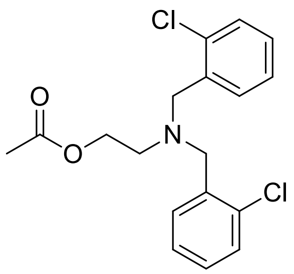

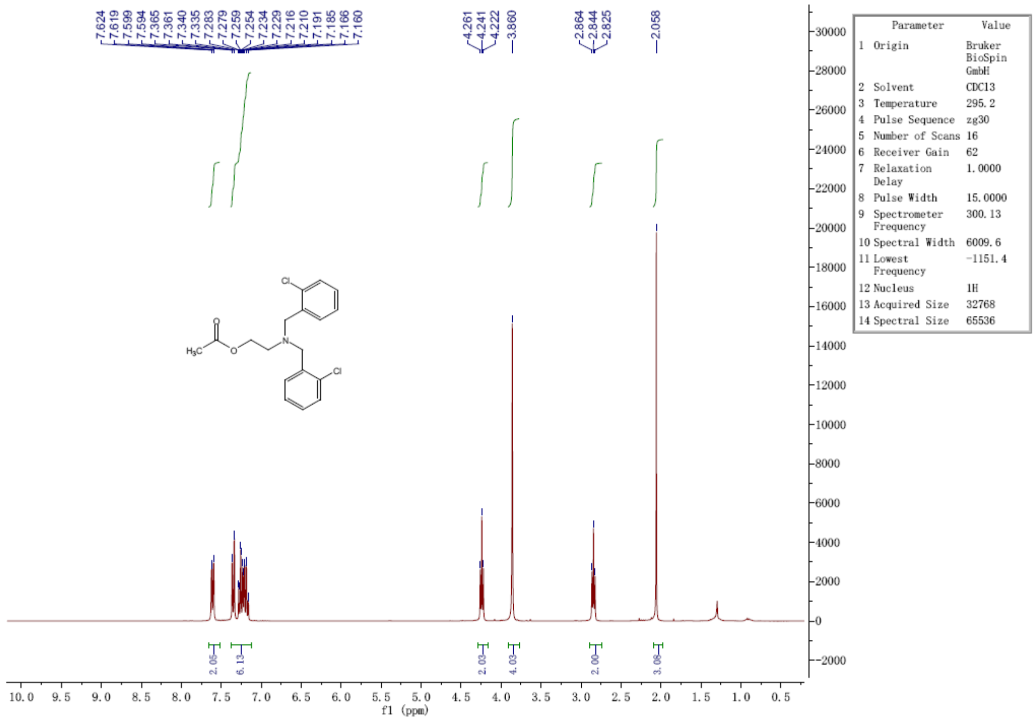

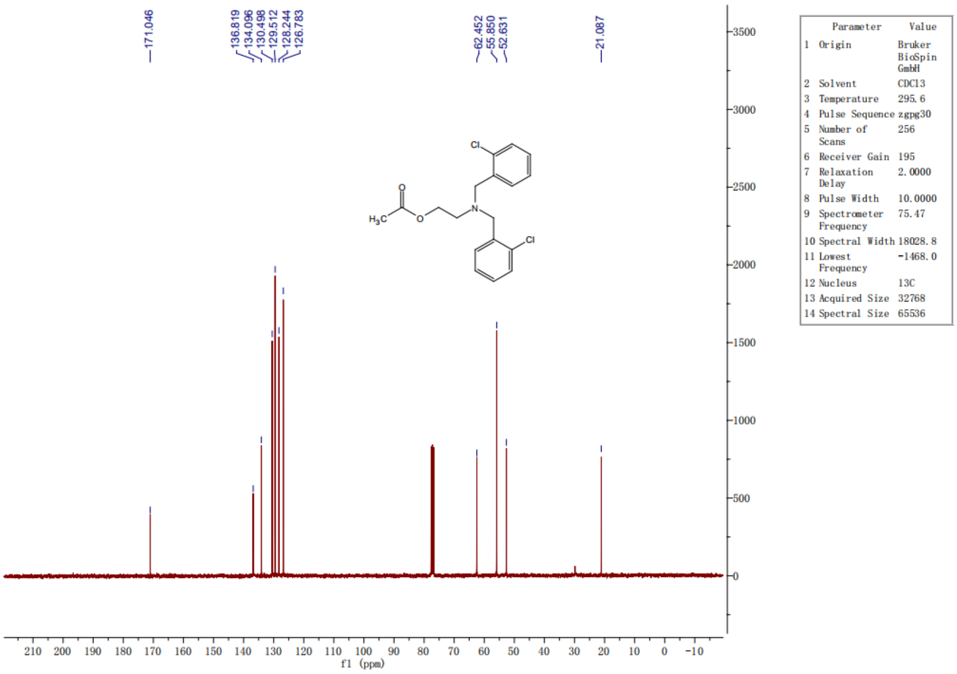

2-(Diallylamino)ethyl acetate (3h)

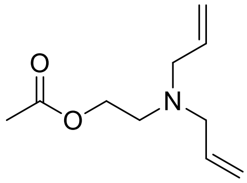

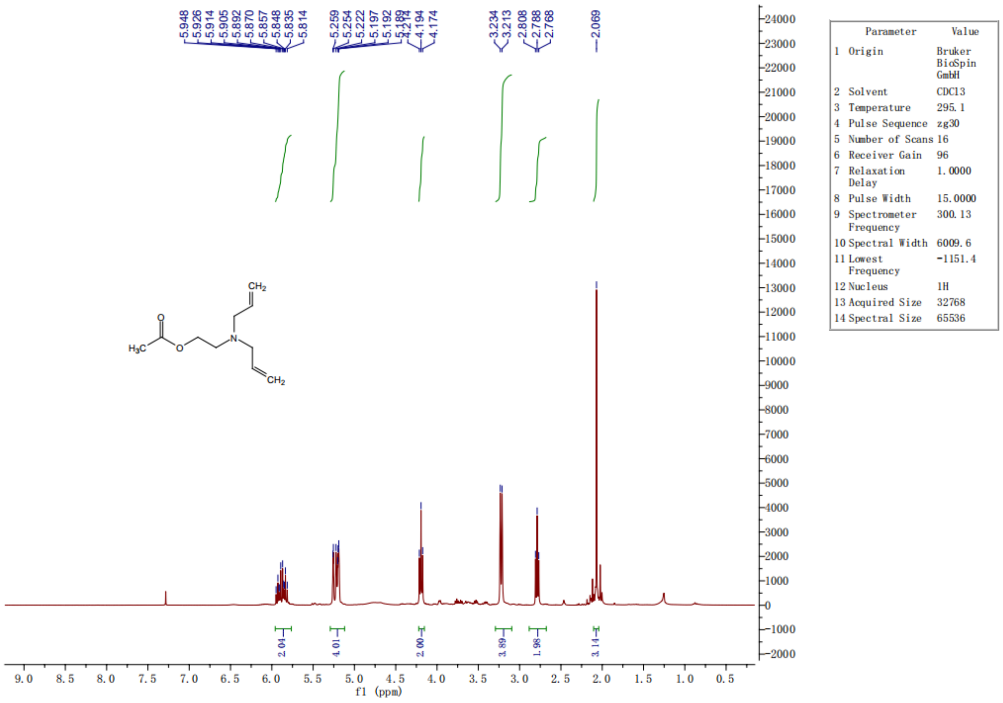

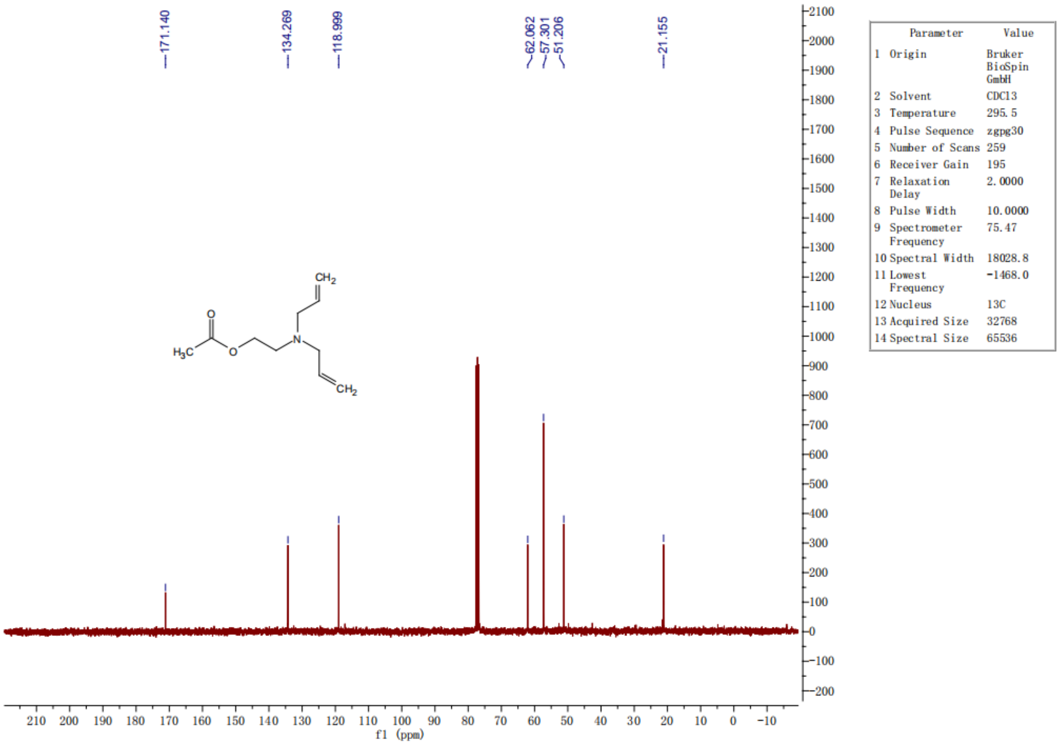

(2*E*,2'*E*)-Dimethyl 4,4'-((2-acetoxyethyl)azanediyl)bis(but-2-enoate) (3i)

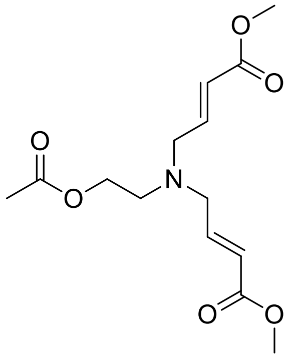

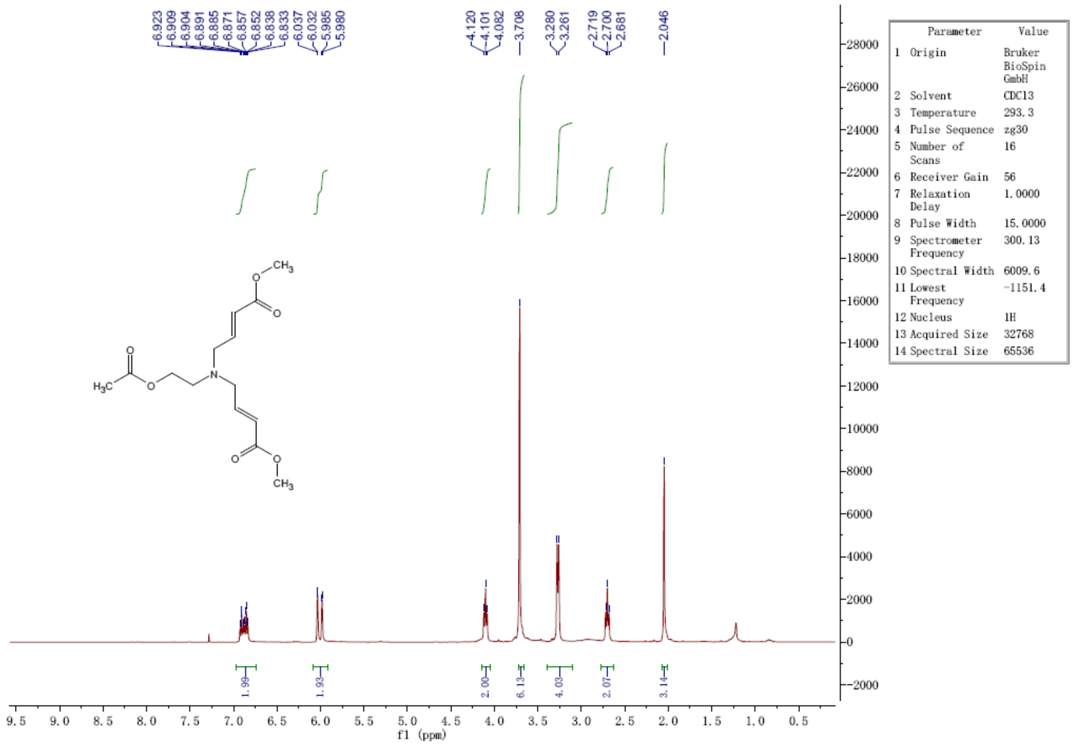

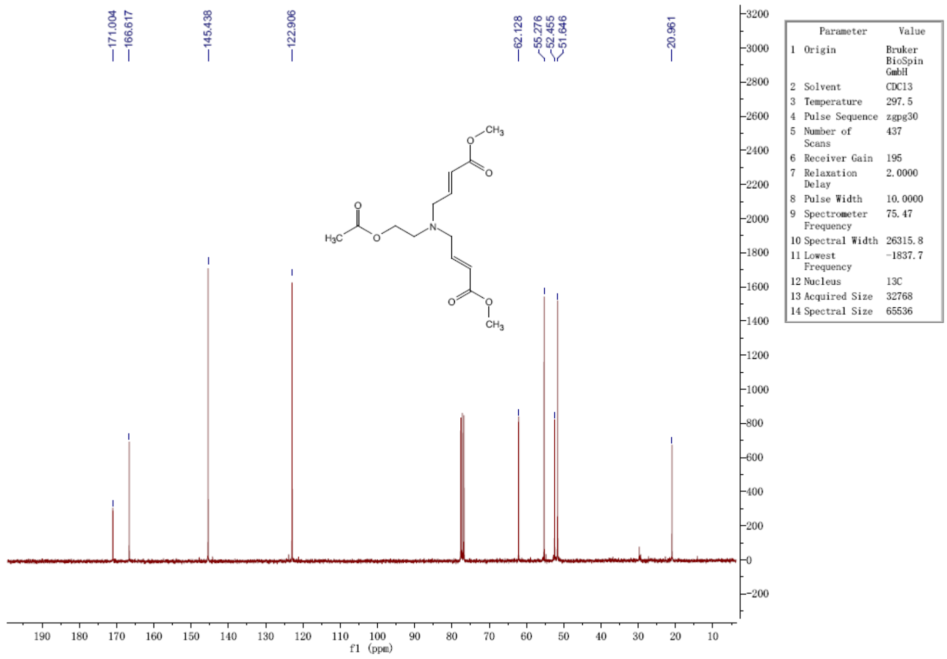

(2*E*,2'*E*)-Diethyl 4,4'-((2-acetoxyethyl)azanediyl)bis(but-2-enoate) (3j)

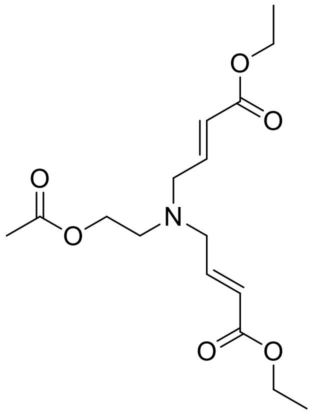

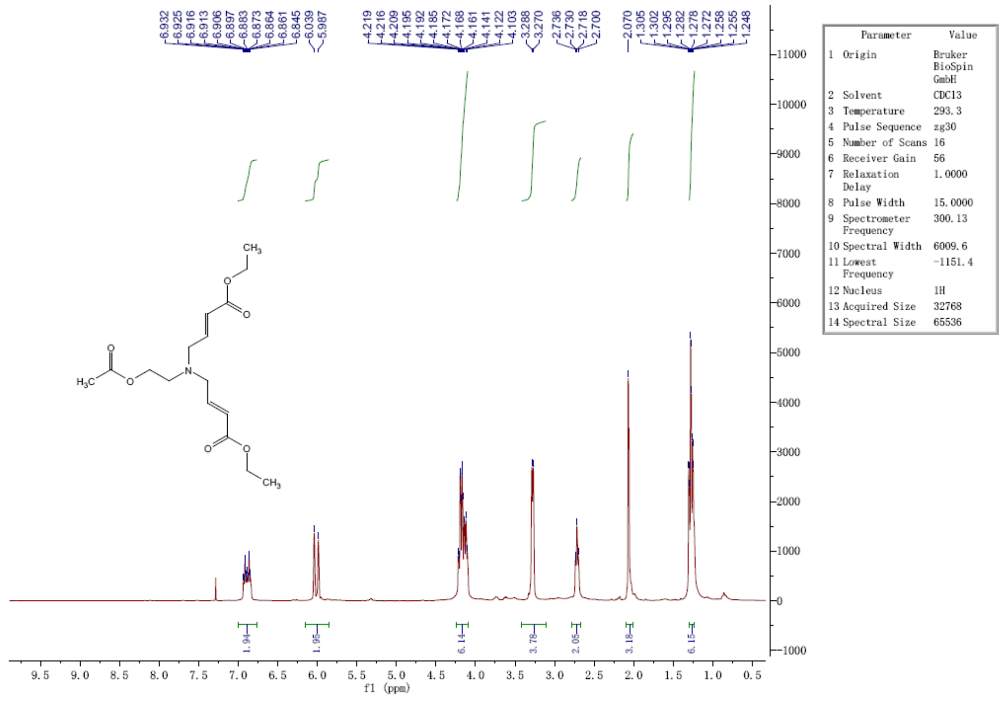

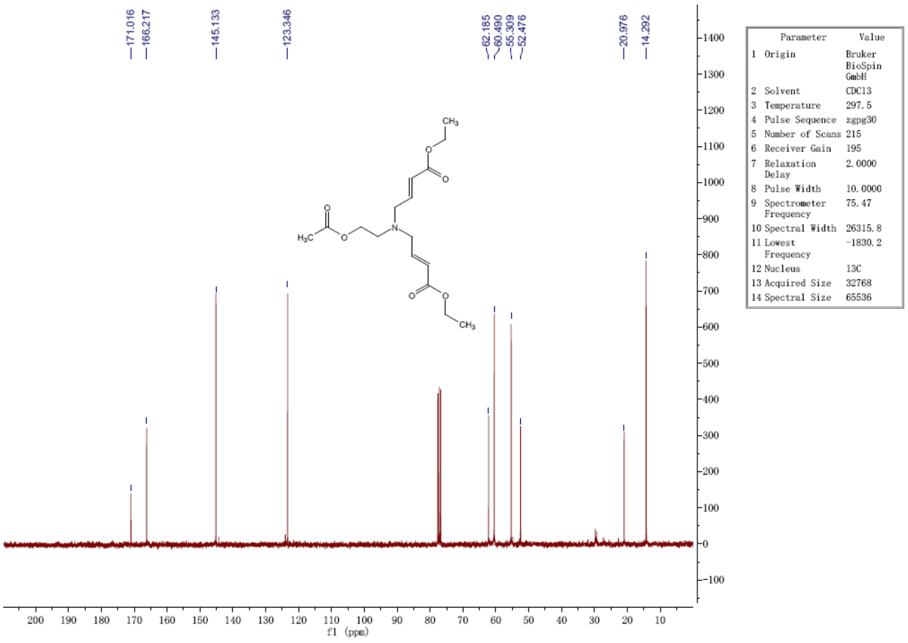

2-(Bis((5-chlorothiophen-2-yl)methyl)amino)ethyl acetate (3k)

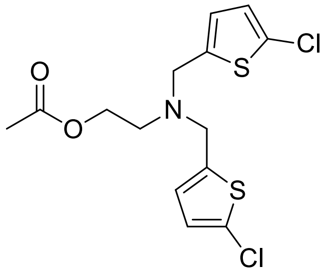

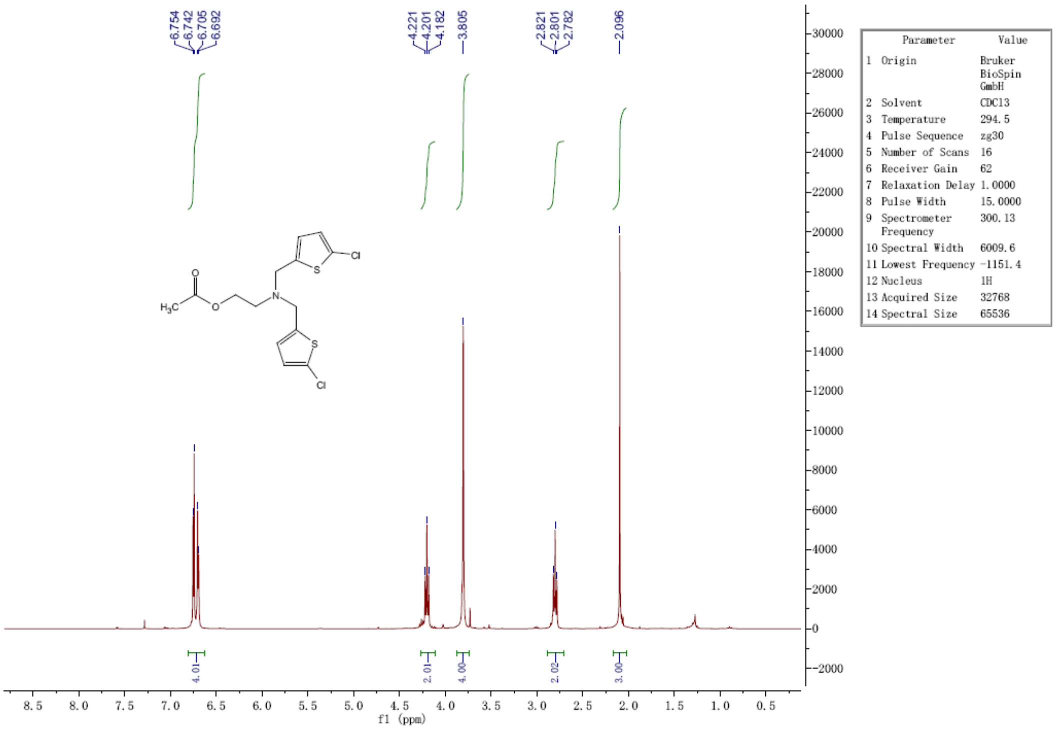

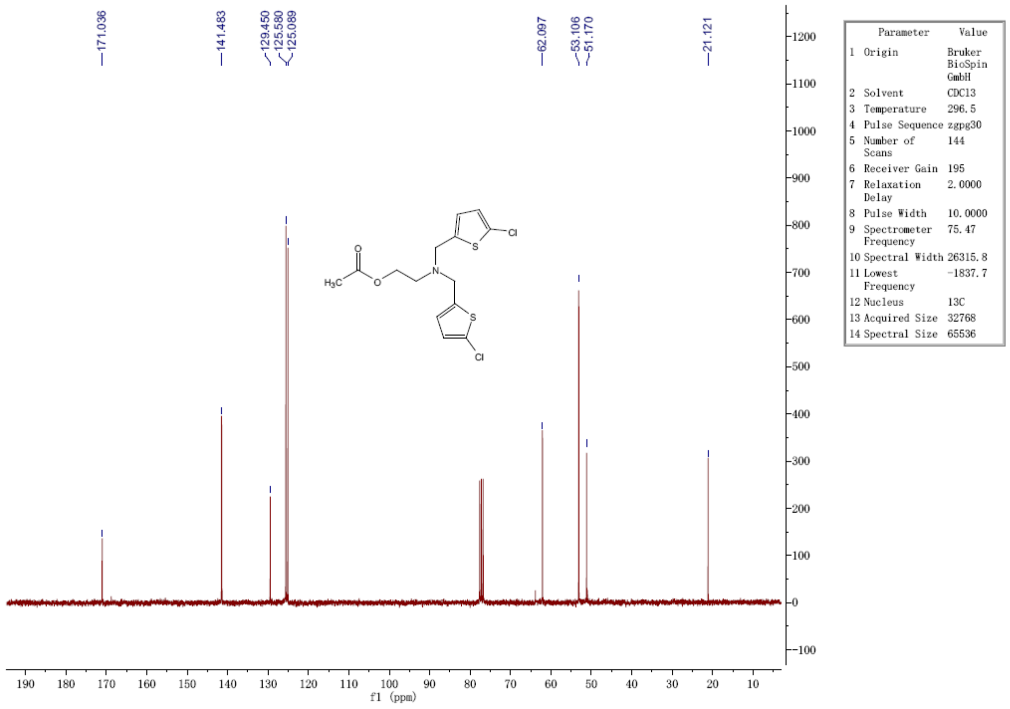

2-(Bis(4-methylbenzyl)amino)-2-methylpropyl acetate (3l)

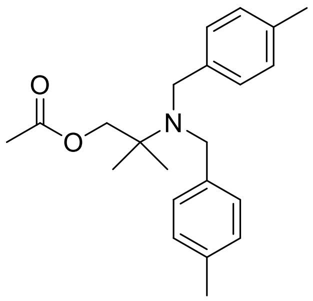

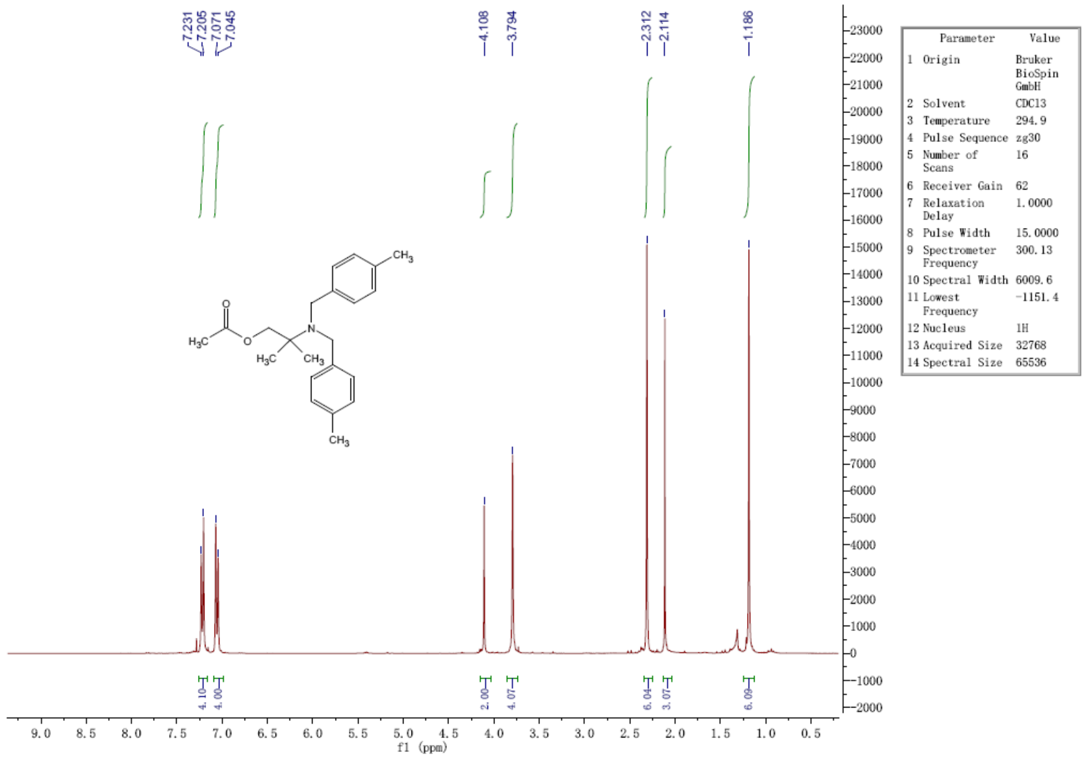

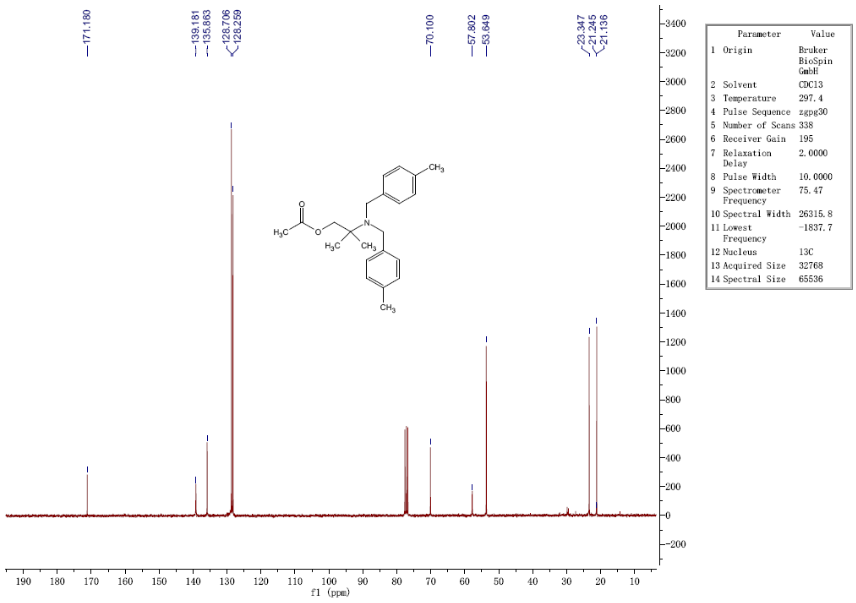

3-Benzylthiazolidin-2-one (5a)

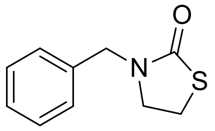

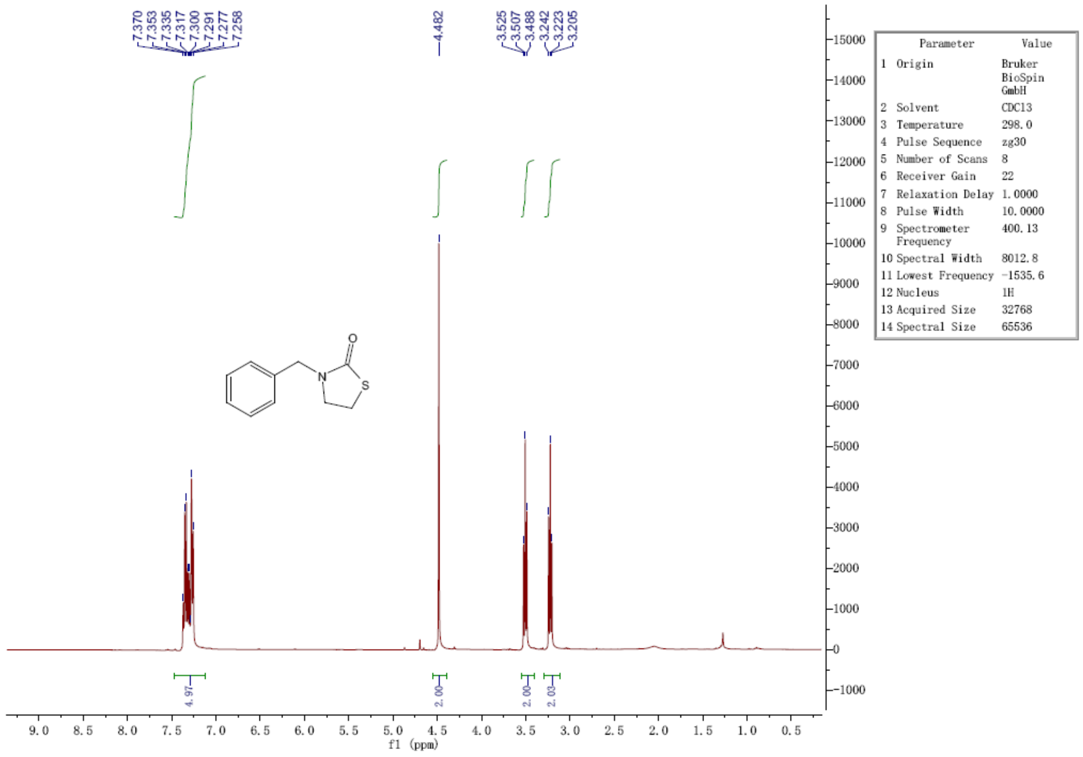

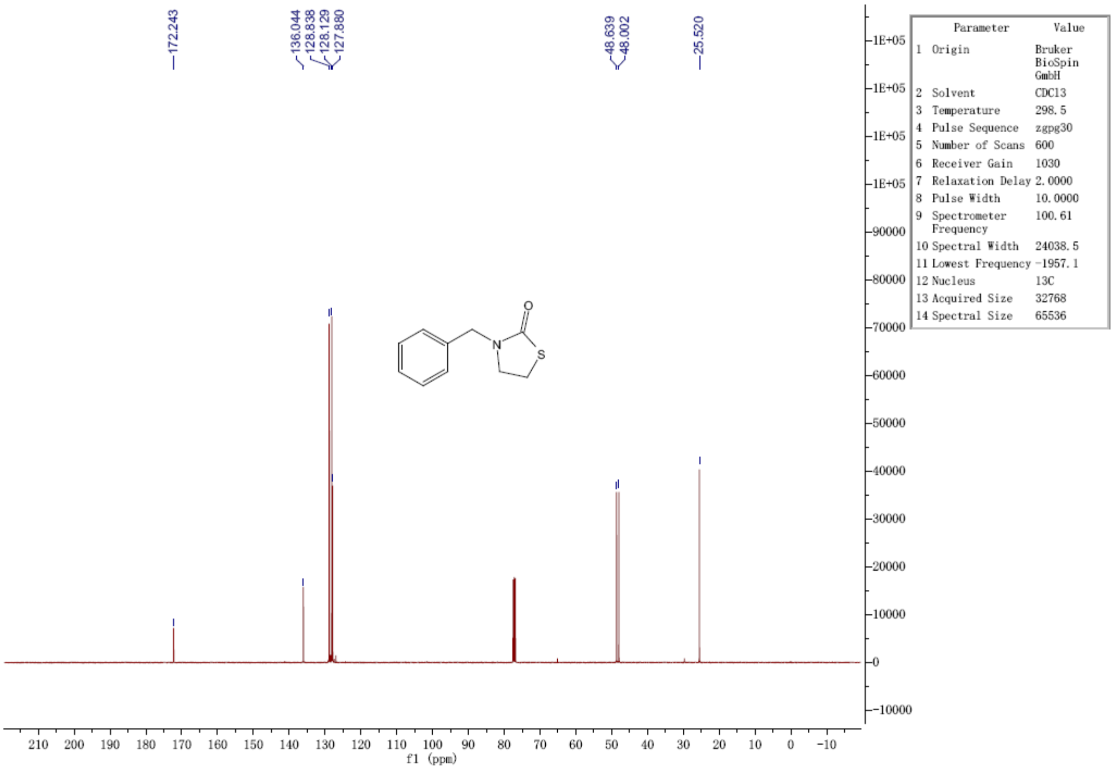

3-(4-Methylbenzyl)thiazolidin-2-one (5b)

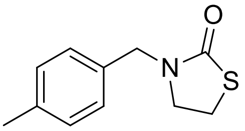

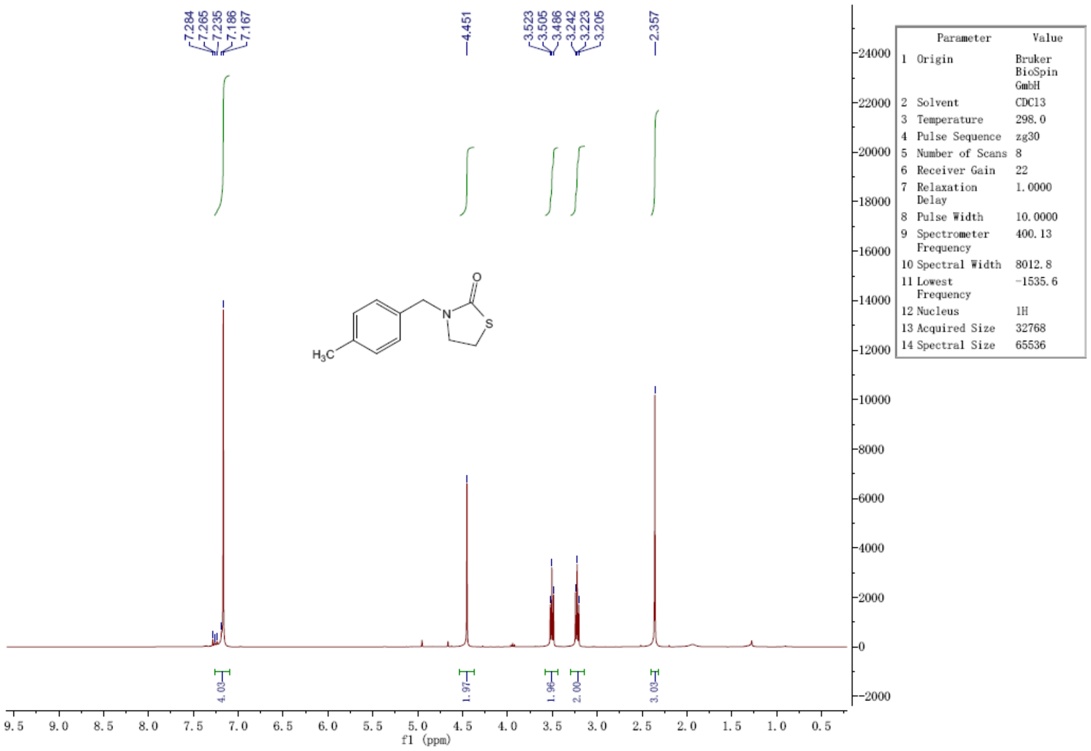

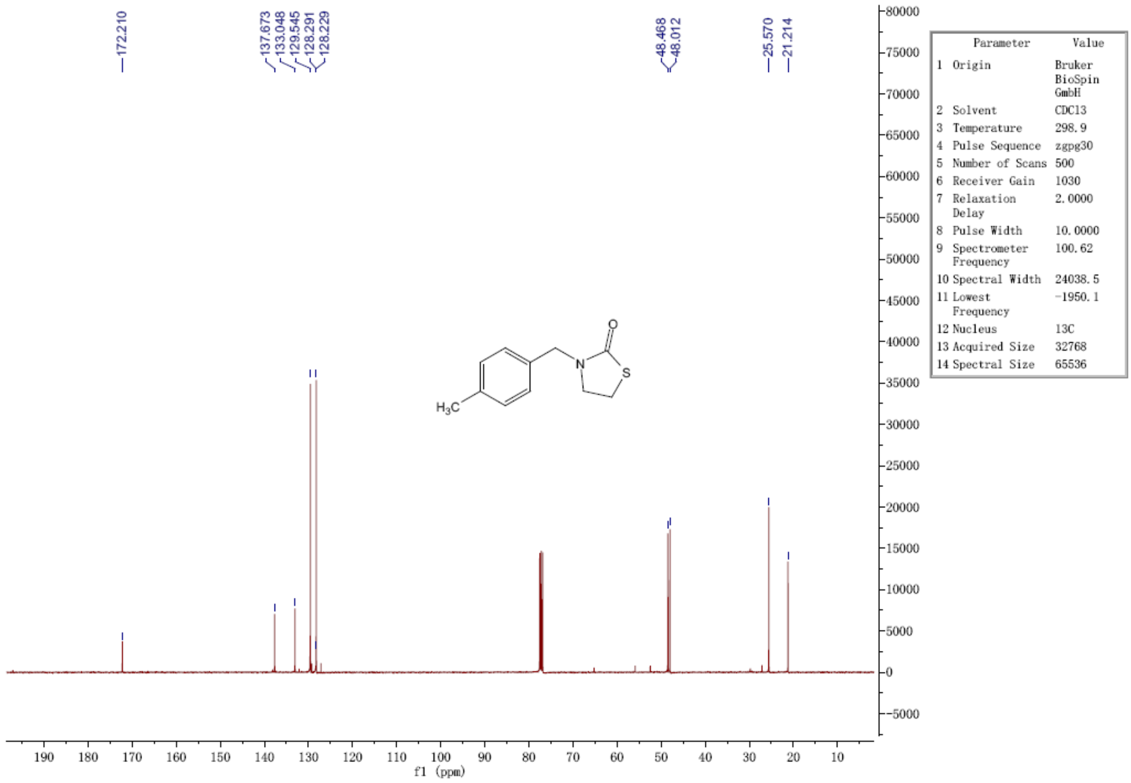

3-(2-Methylbenzyl)thiazolidin-2-one (5c)

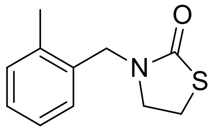

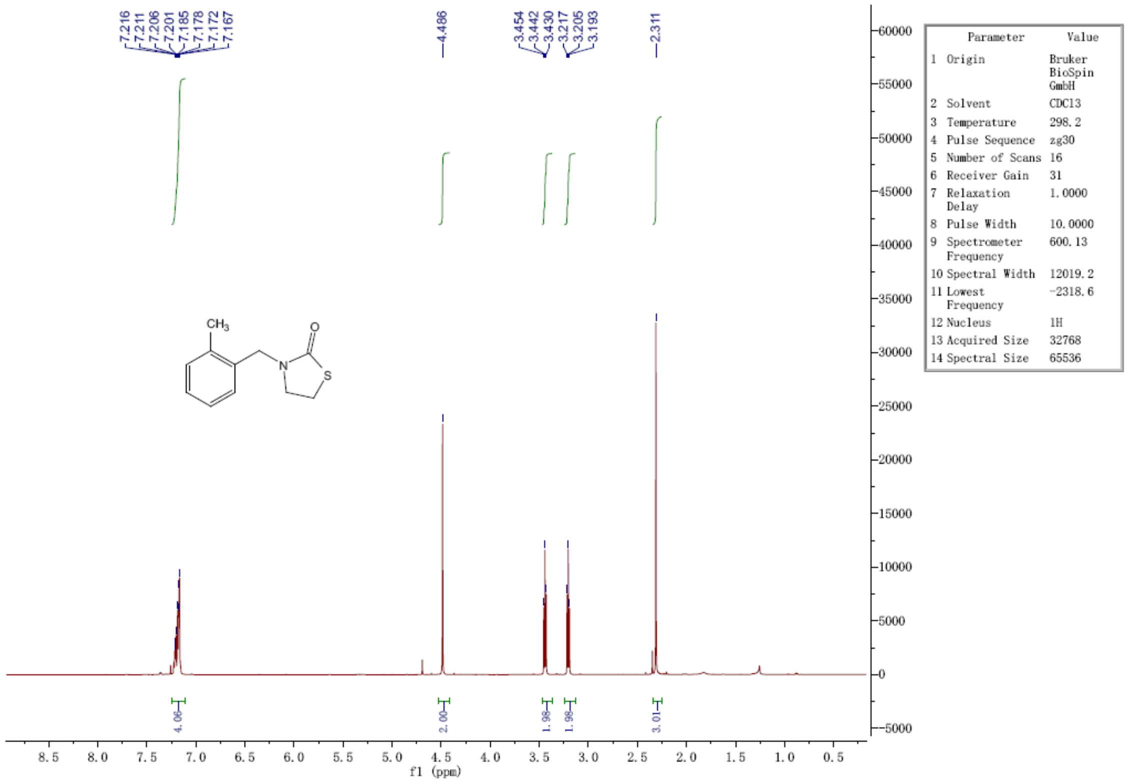

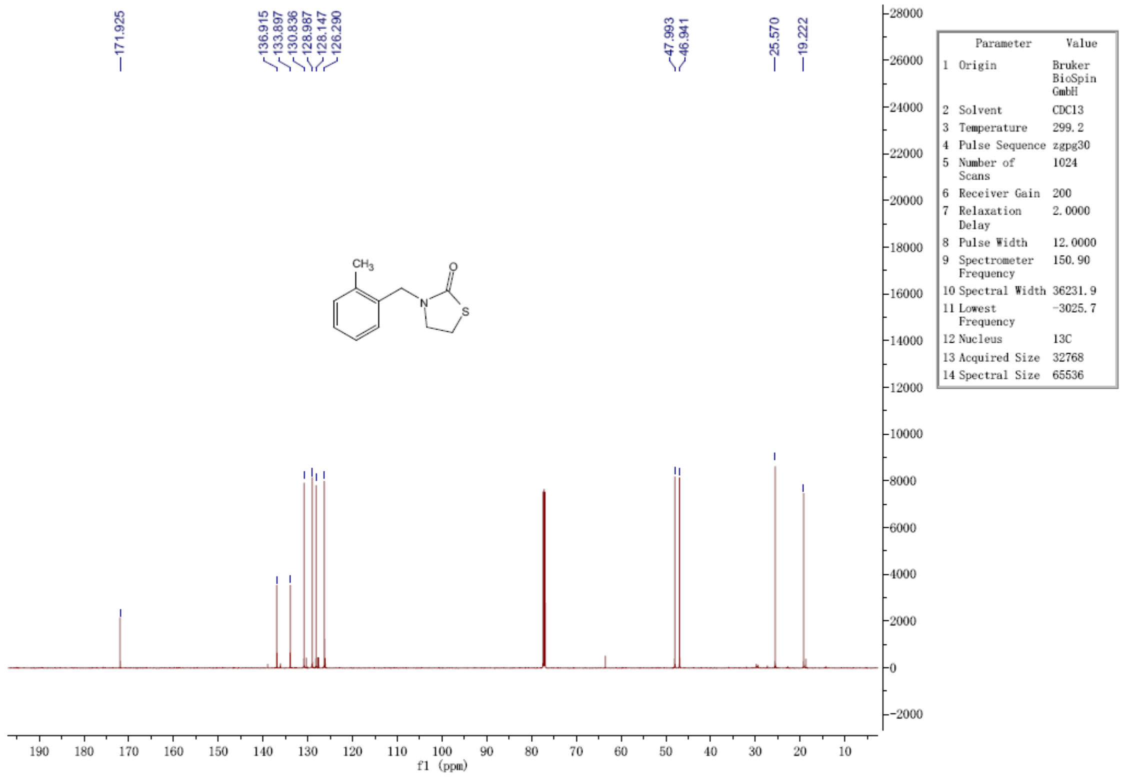

3-(4-(*tert*-Butyl)benzyl)thiazolidin-2-one (5d)

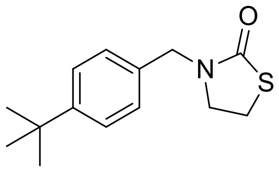

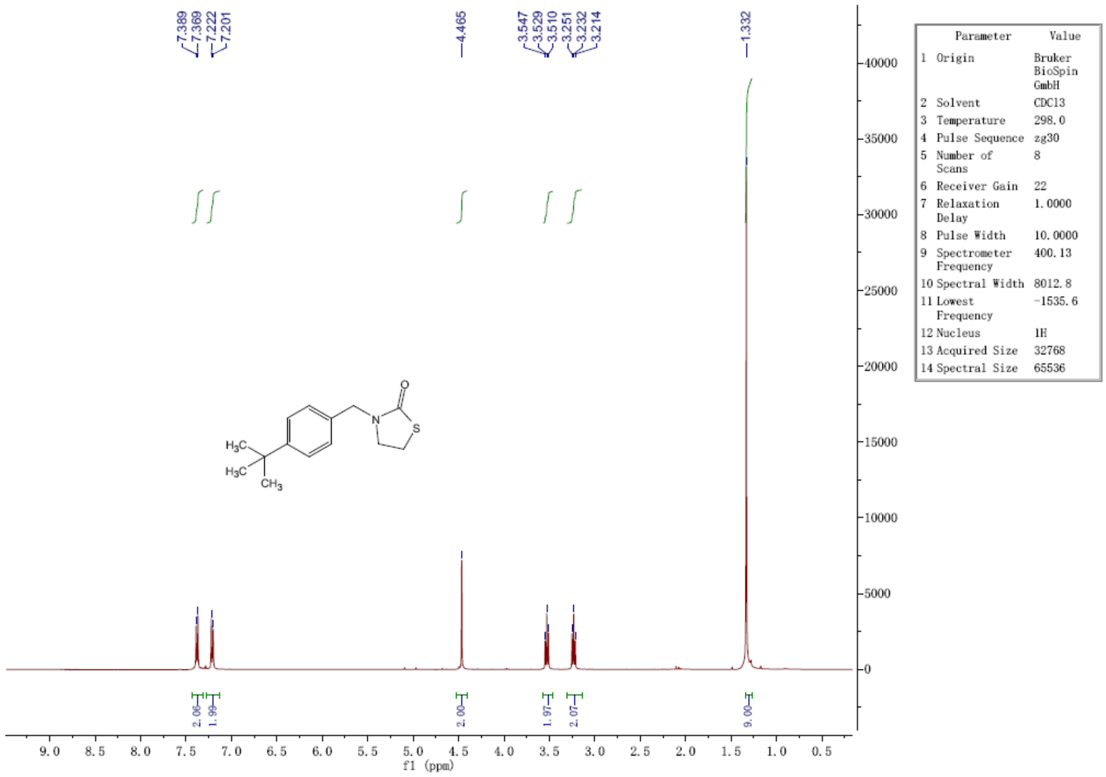

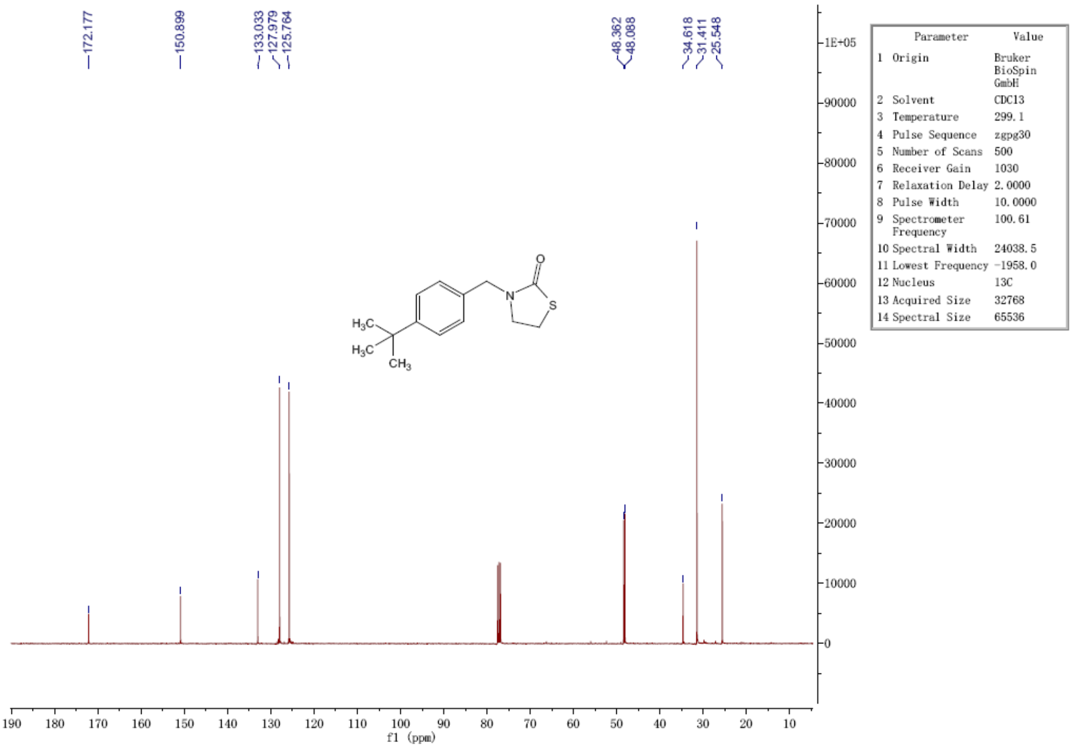

3-(4-Bromobenzyl)thiazolidin-2-one (5e)

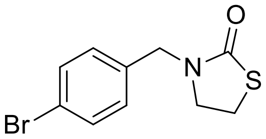

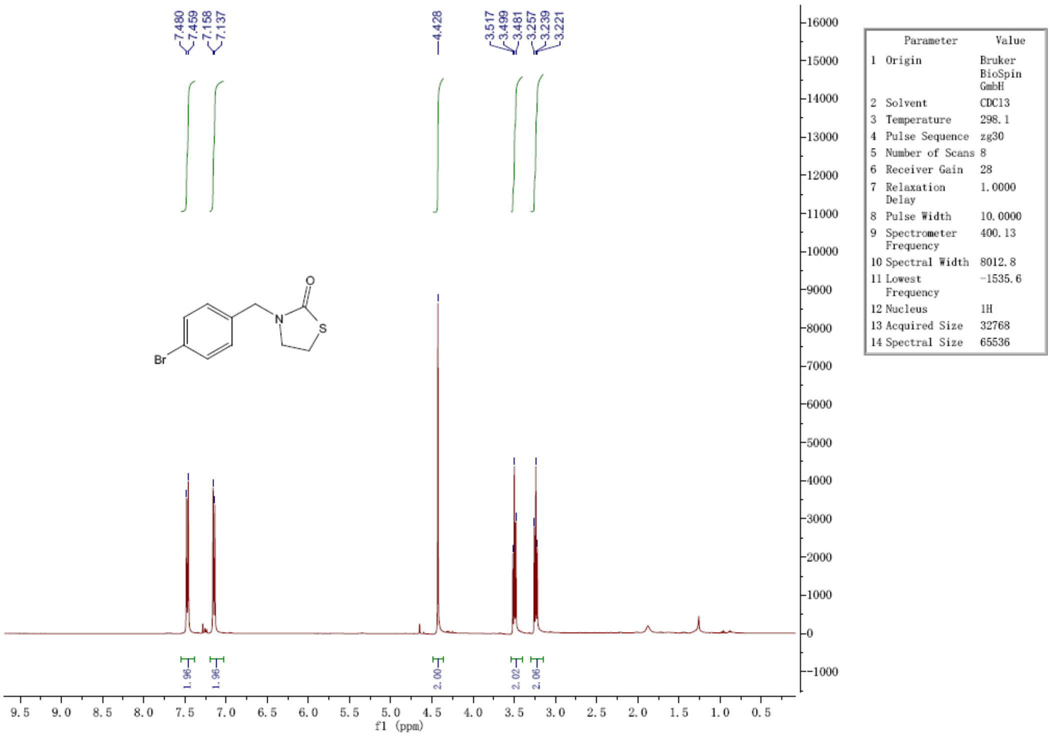

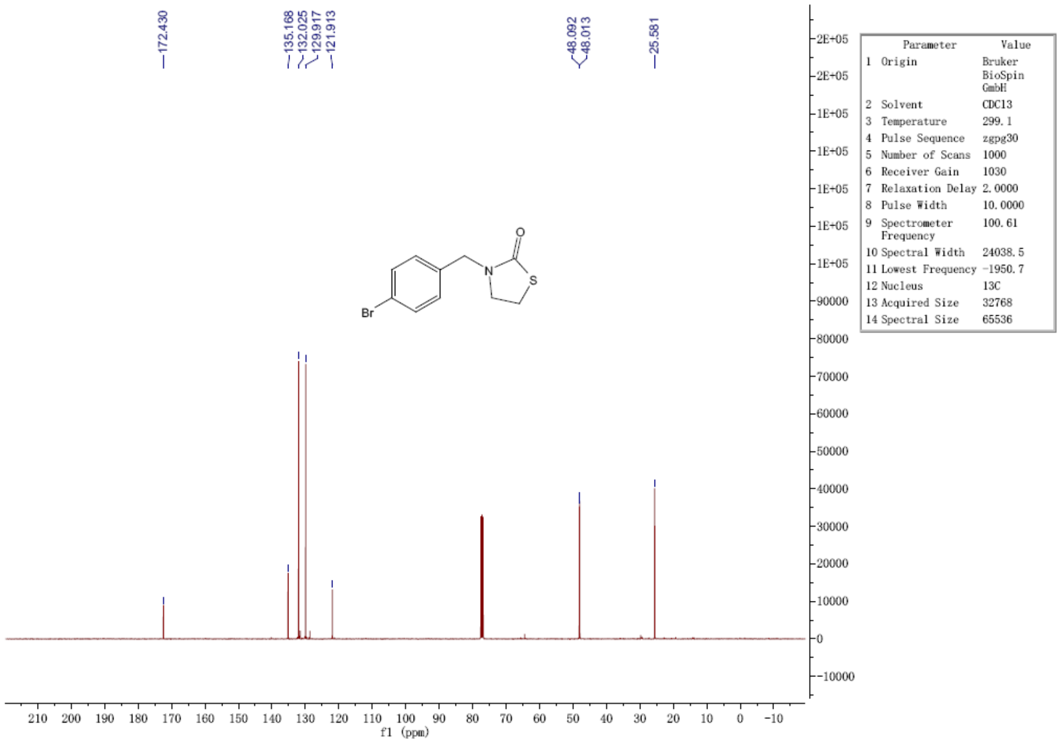

3-(2-Chlorobenzyl)thiazolidin-2-one (5f)

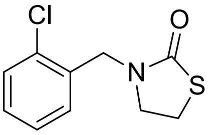

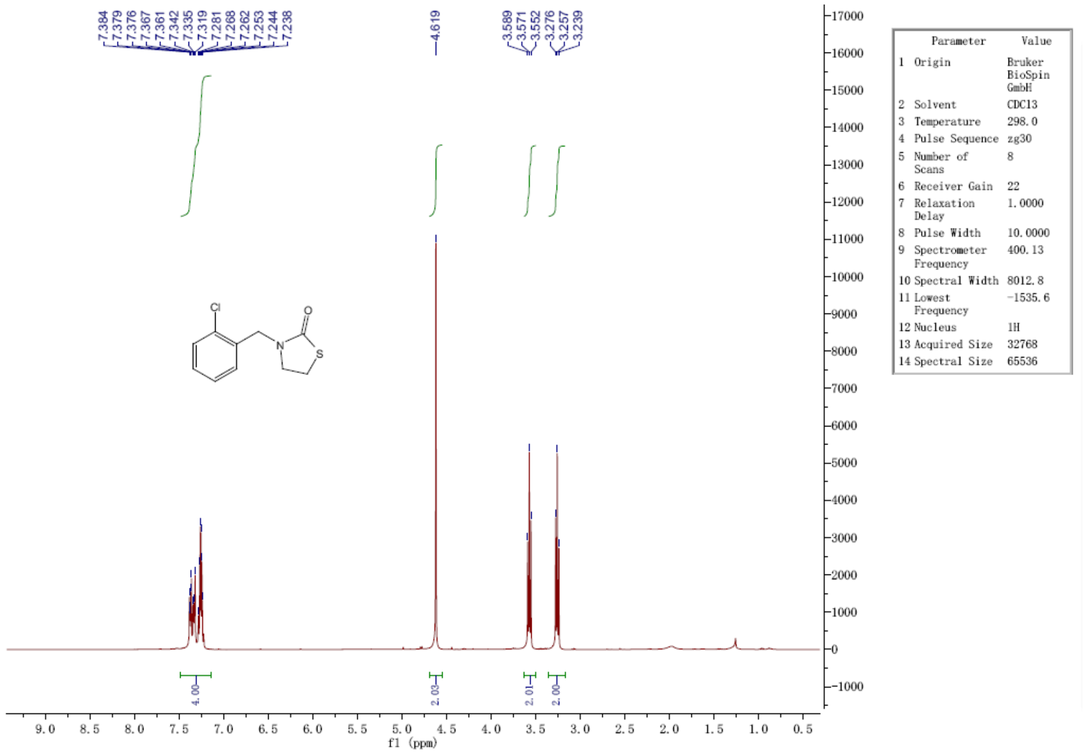

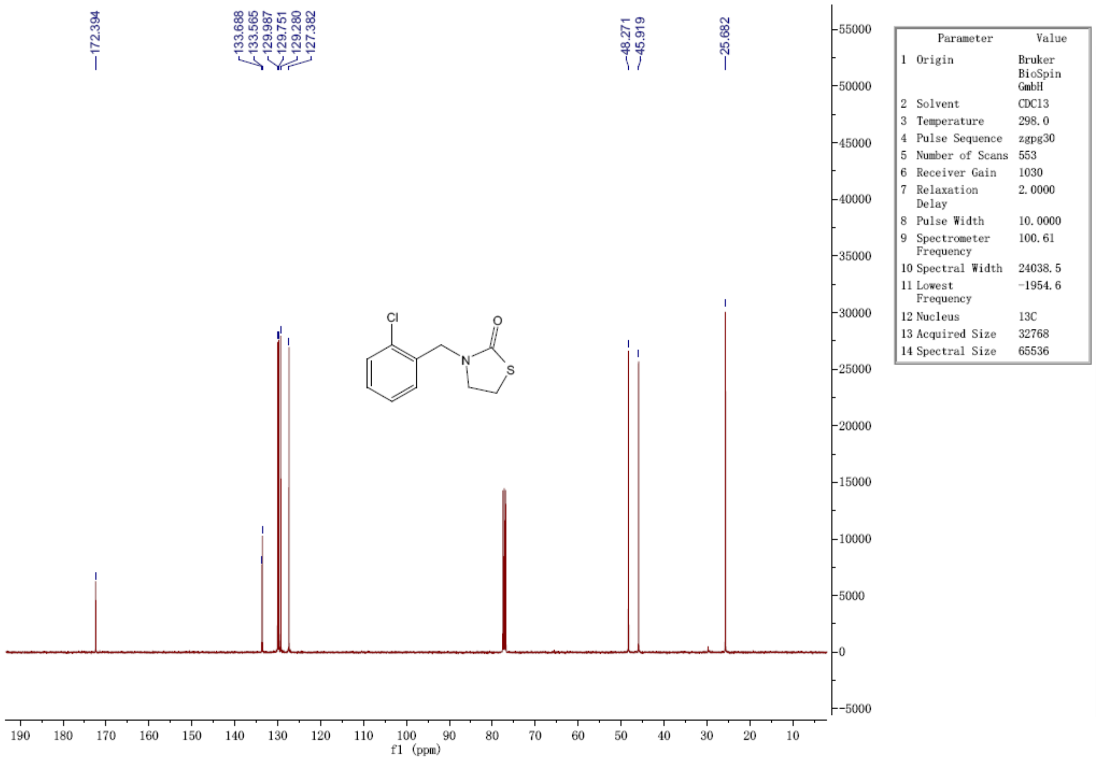

3-(4-Fluorobenzyl)thiazolidin-2-one (5g)

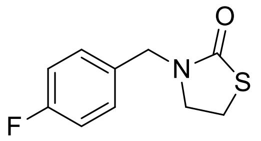

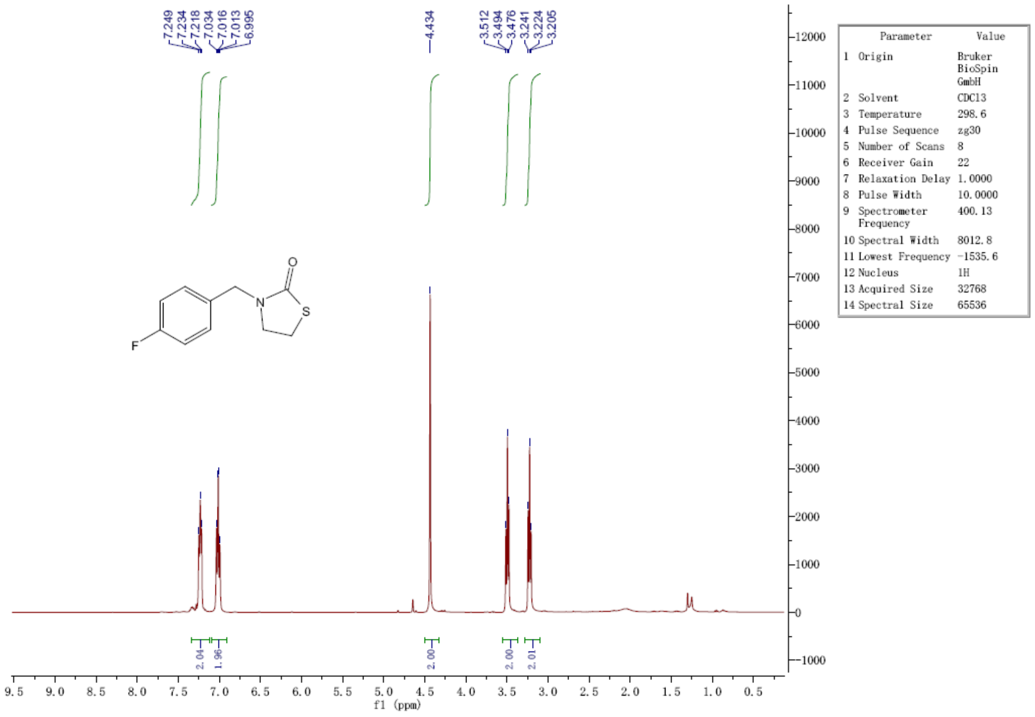

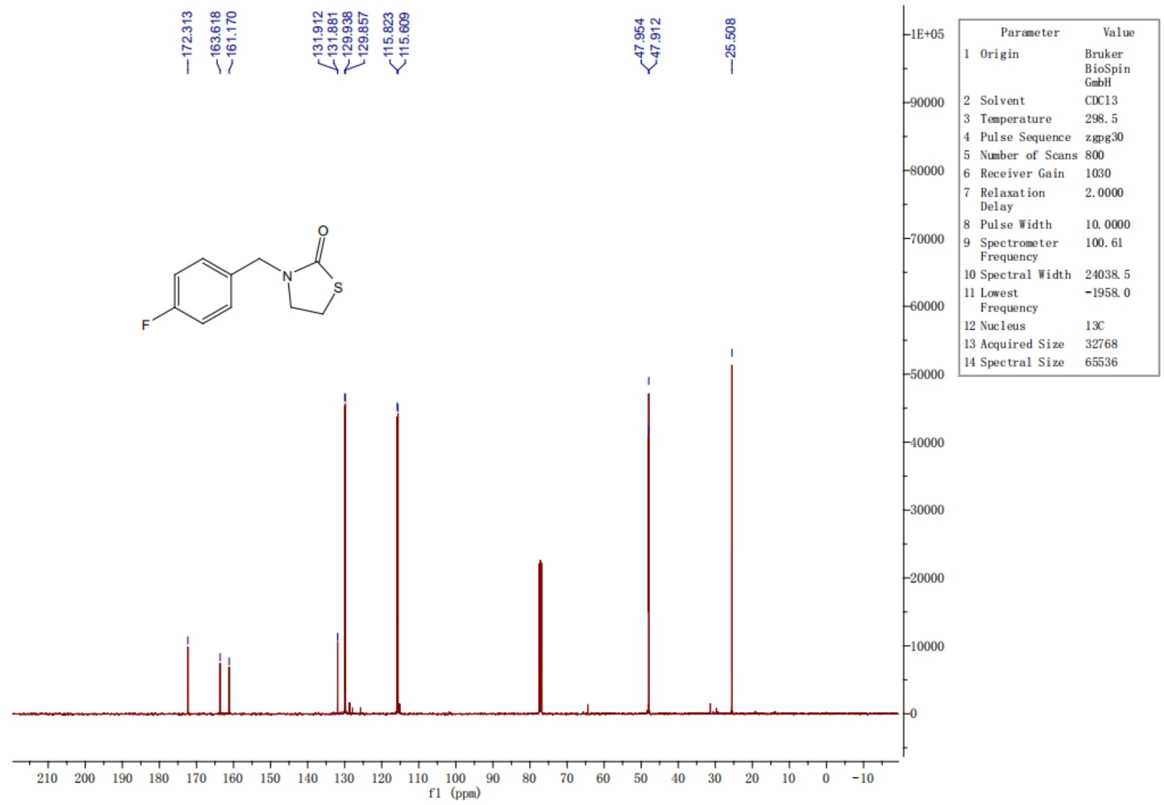

3-(2-Fluorobenzyl)thiazolidin-2-one (5h)

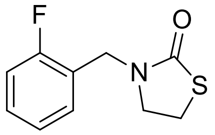

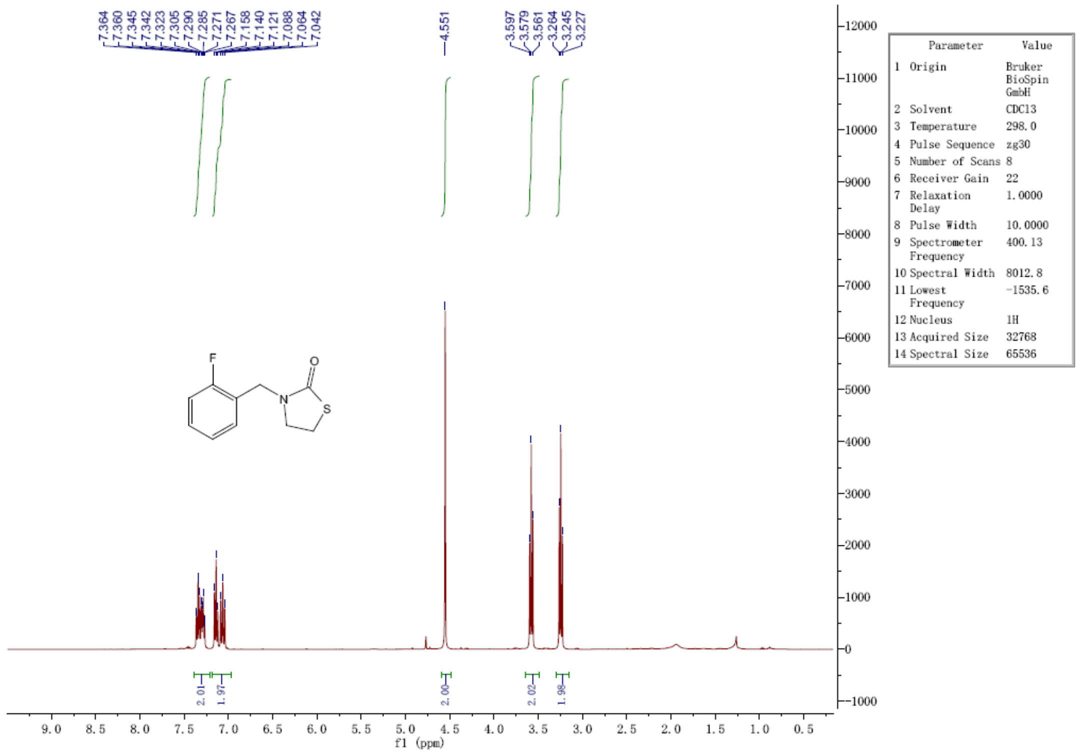

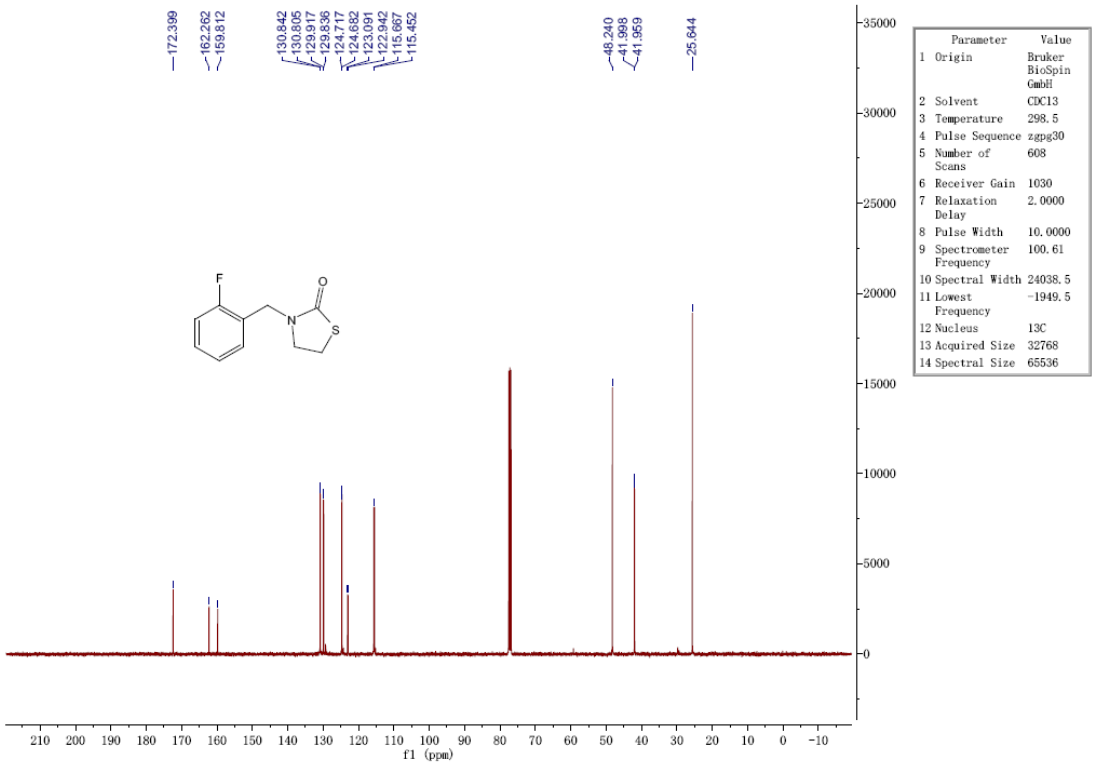

4-((2-Oxothiazolidin-3-yl)methyl)benzonitrile (5i)

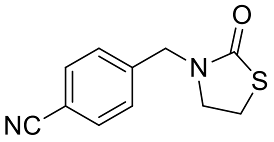

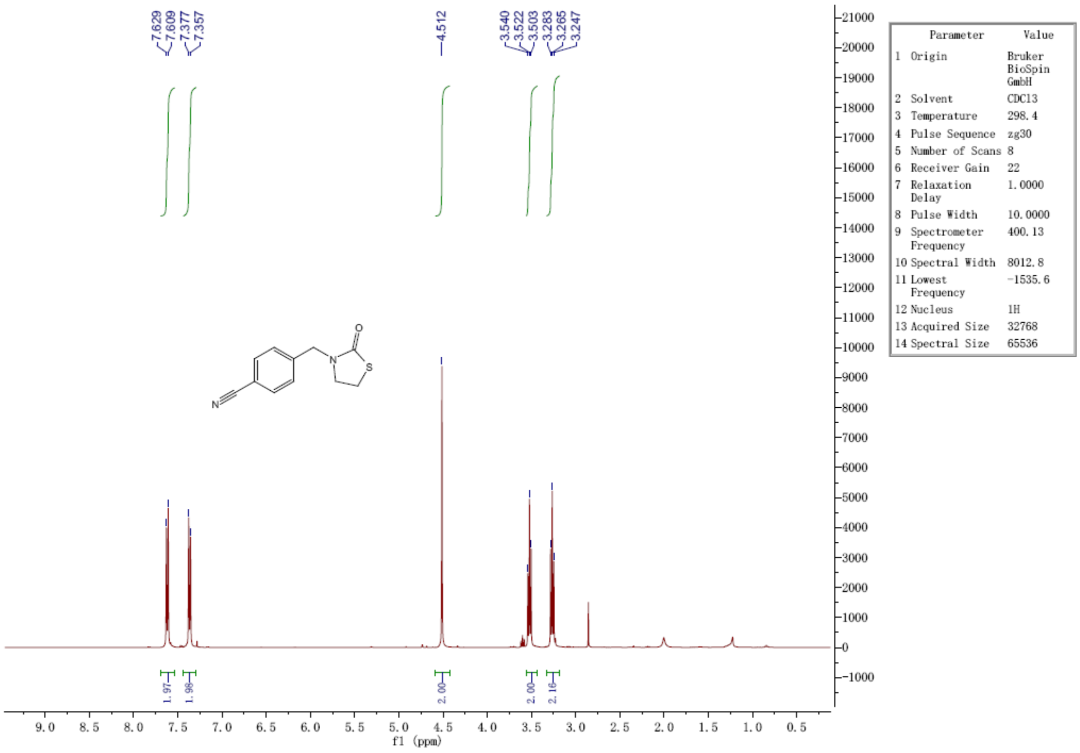

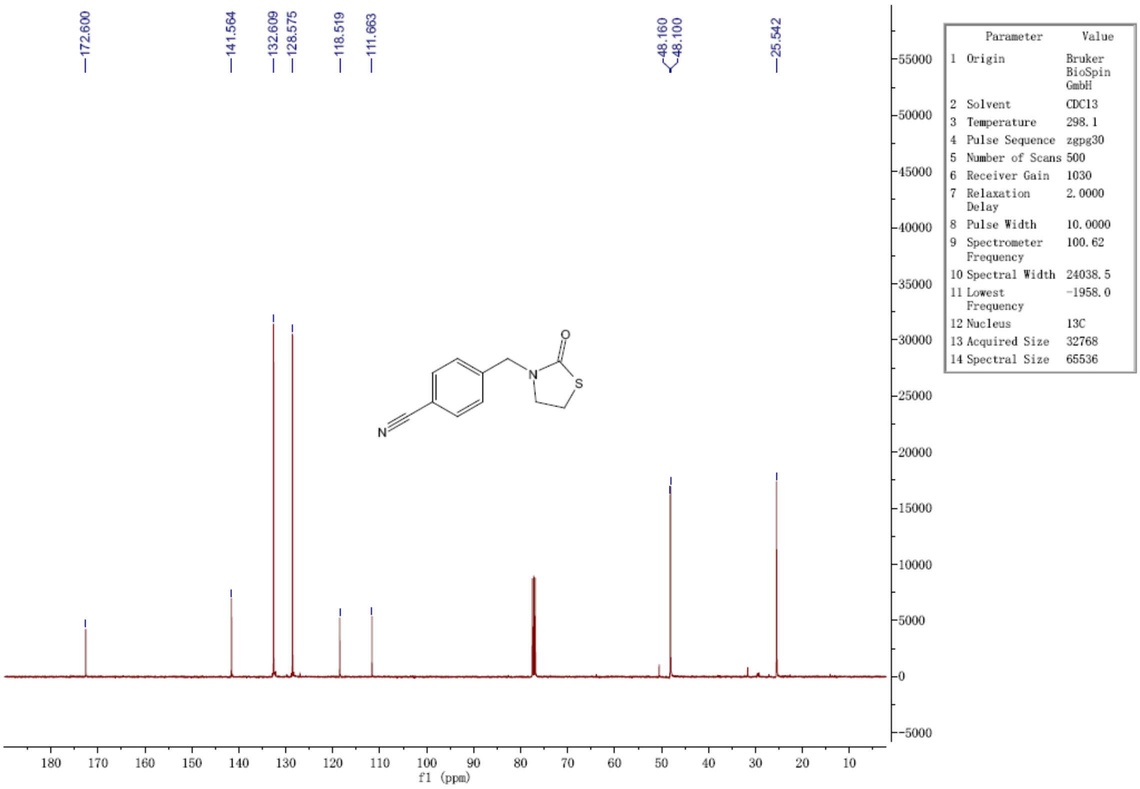

3-((6-Methylpyridin-2-yl)methyl)thiazolidin-2-one (5j)

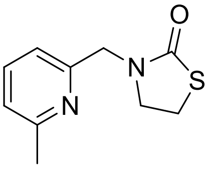

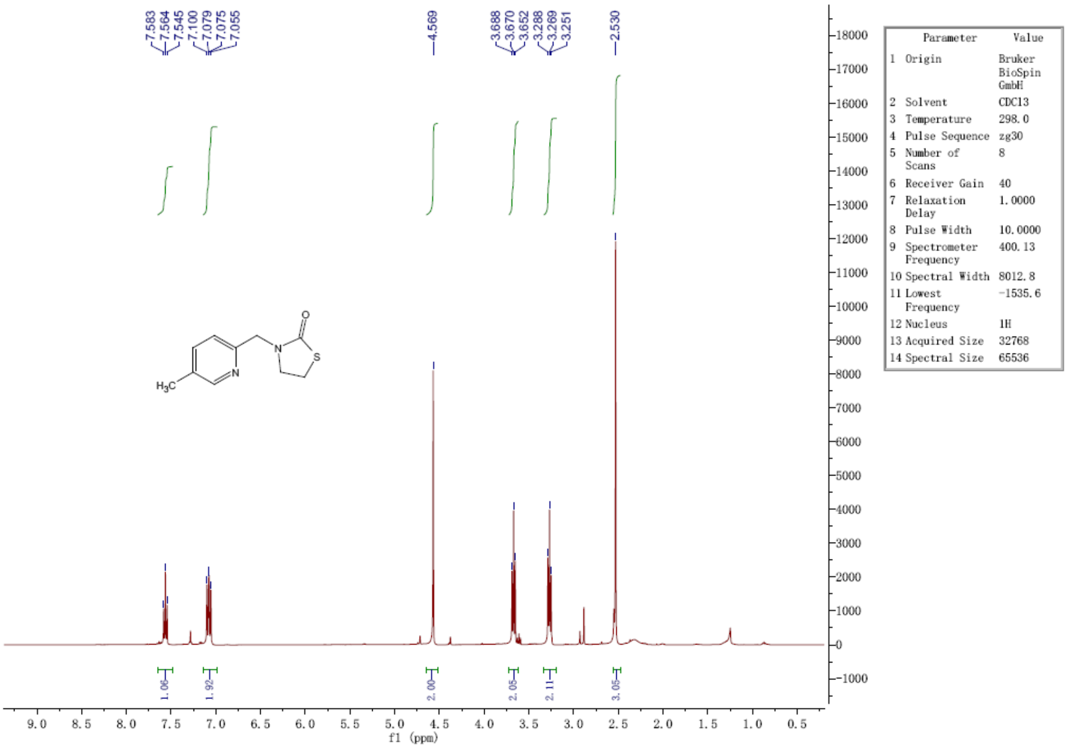

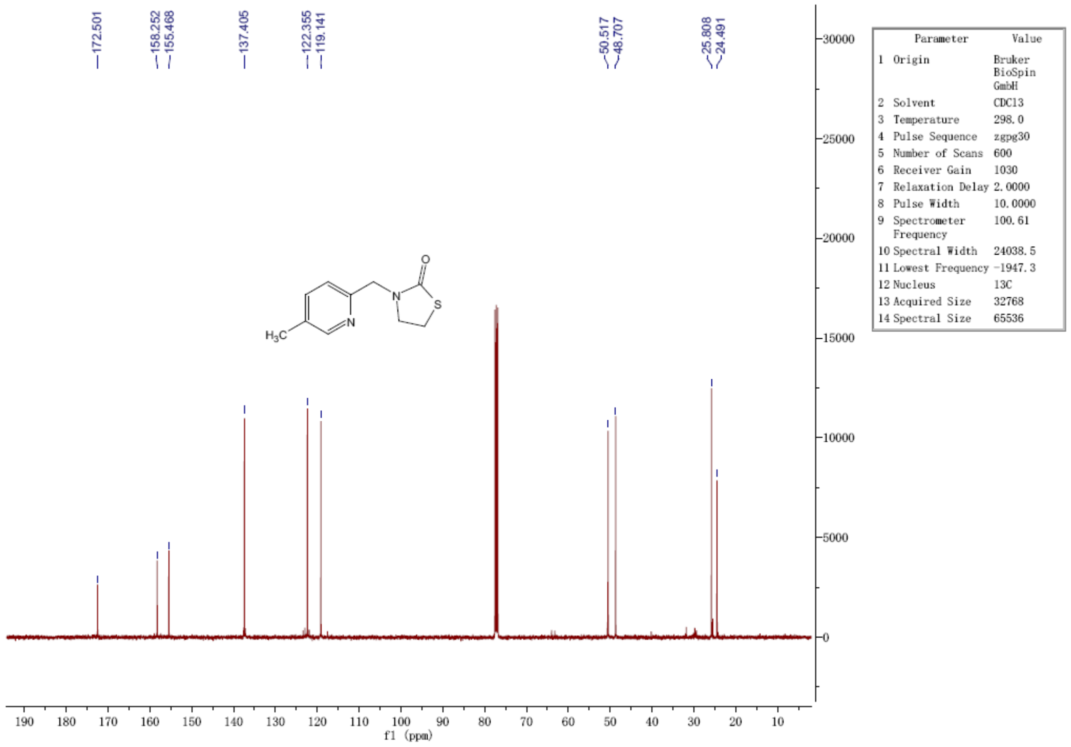

3-((5-Chlorothiophen-2-yl)methyl)thiazolidin-2-one (5k)

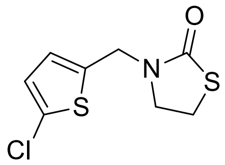

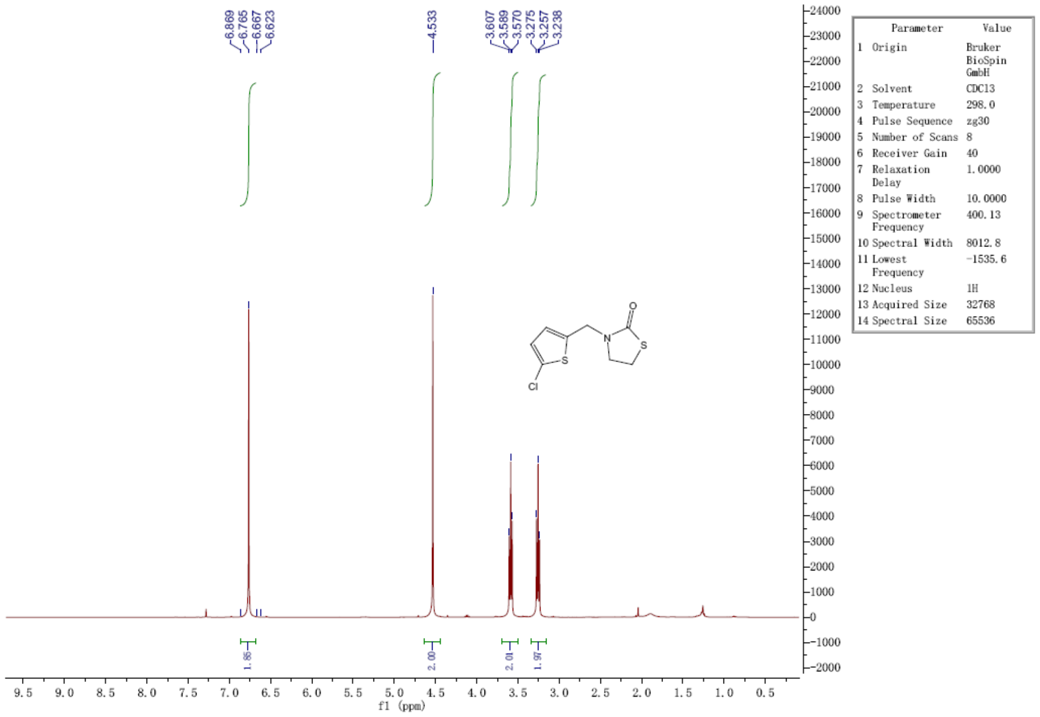

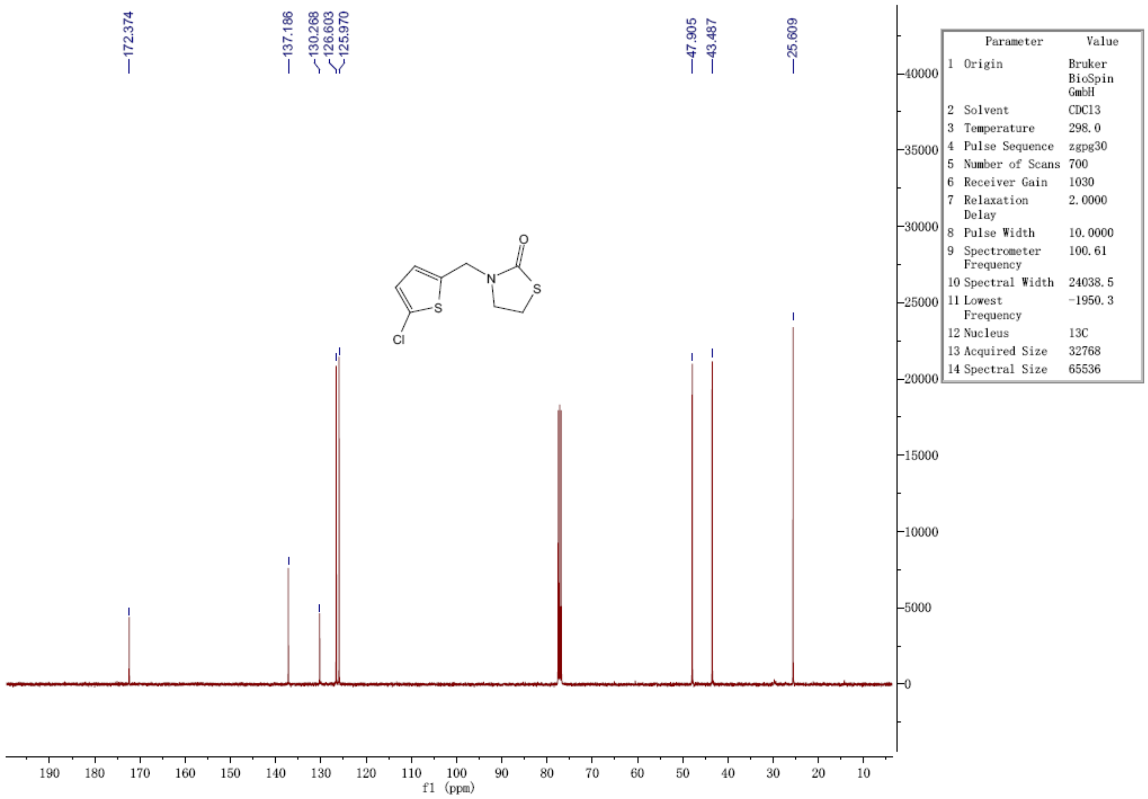

2-(Dibenzylamino)ethanol (6)

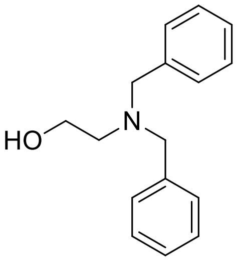

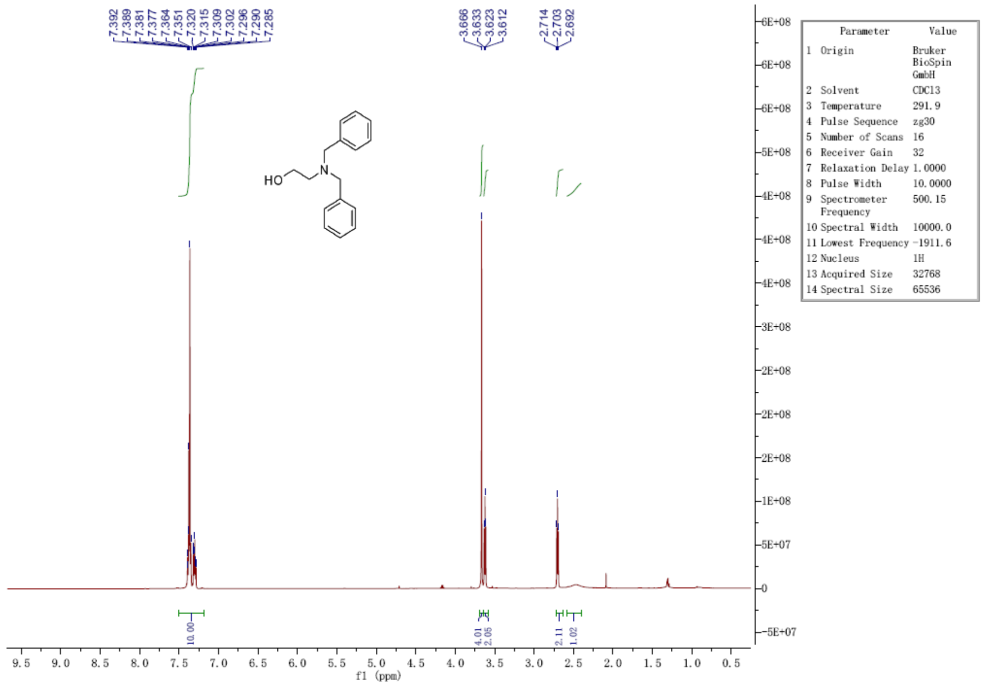

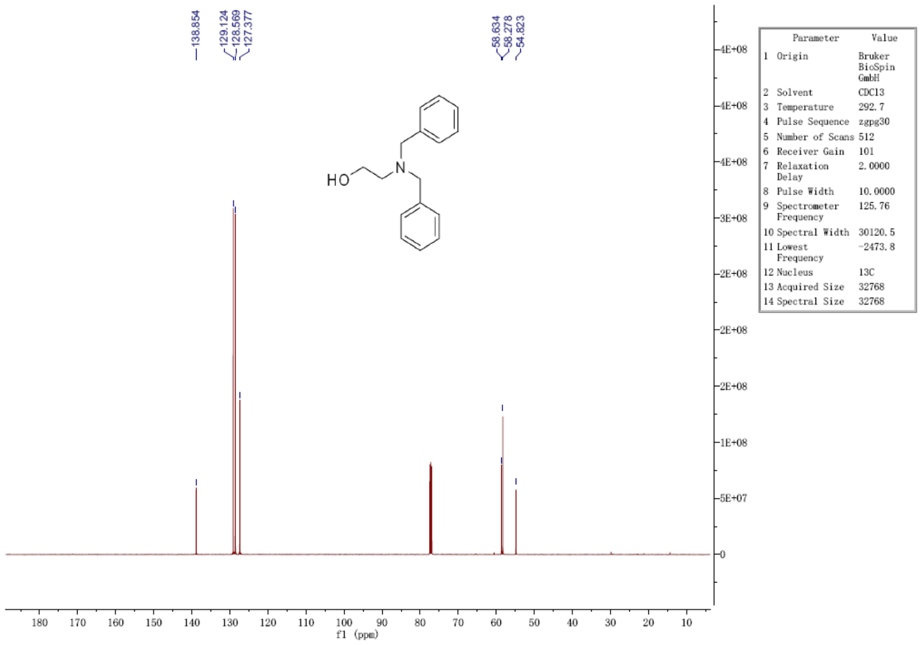

Supplement: File 1 — Characterization data and copies of NMR spectra. [file Beilstein_J_Org_Chem-16-492-s001.pdf]
